# A modeling-based approach to optimize COVID-19 vaccine dosing schedules for improved protection

**DOI:** 10.1172/jci.insight.169860

**Published:** 2023-07-10

**Authors:** Prashant Dogra, Carmine Schiavone, Zhihui Wang, Javier Ruiz-Ramírez, Sergio Caserta, Daniela I. Staquicini, Christopher Markosian, Jin Wang, H. Dirk Sostman, Renata Pasqualini, Wadih Arap, Vittorio Cristini

**Affiliations:** 1 Mathematics in Medicine Program, Department of Medicine, Houston Methodist Research Institute, Houston, Texas, USA.; 2 Department of Physiology and Biophysics, Weill Cornell Medical College, New York, New York, USA.; 3 Department of Chemical, Materials and Industrial Production Engineering, University of Naples Federico II, Naples, Italy.; 4 Neal Cancer Center, Houston Methodist Research Institute, Houston, Texas, USA.; 5 Centro de Ciencias de la Salud, Universidad Autónoma de Aguascalientes, Aguascalientes, Mexico.; 6 CEINGE Advanced Biotechnologies, Naples, Italy.; 7 Rutgers Cancer Institute of New Jersey, Newark, New Jersey, USA.; 8 Division of Cancer Biology, Department of Radiation Oncology, Rutgers New Jersey Medical School, Newark, New Jersey, USA.; 9 Immunobiology and Transplant Science Center, Department of Surgery, Houston Methodist Research Institute, Houston, Texas, USA.; 10 Department of Surgery, Weill Cornell Medical College, Cornell University, New York, New York, USA.; 11 Weill Cornell Medicine, New York, New York, USA.; 12 Houston Methodist Research Institute, Houston, Texas, USA.; 13 Houston Methodist Academic Institute, Houston, Texas, USA.; 14 Division of Hematology/Oncology, Department of Medicine, Rutgers New Jersey Medical School, Newark, New Jersey, USA.; 15 Department of Imaging Physics, University of Texas MD Anderson Cancer Center, Houston, Texas, USA.; 16 Physiology, Biophysics, and Systems Biology Program, Graduate School of Medical Sciences, Weill Cornell Medicine, New York, New York, USA.

**Keywords:** COVID-19, Vaccines, Adaptive immunity, Antigen presentation, Immunoglobulins

## Abstract

While the development of different vaccines slowed the dissemination of SARS-CoV-2, the occurrence of breakthrough infections has continued to fuel the COVID-19 pandemic. To secure at least partial protection in the majority of the population through 1 dose of a COVID-19 vaccine, *delayed* administration of boosters has been implemented in many countries. However, waning immunity and emergence of new variants of SARS-CoV-2 suggest that such measures may induce breakthrough infections due to intermittent lapses in protection. Optimizing vaccine dosing schedules to ensure prolonged continuity in protection could thus help control the pandemic. We developed a mechanistic model of immune response to vaccines as an in silico tool for dosing schedule optimization. The model was calibrated with clinical data sets of acquired immunity to COVID-19 mRNA vaccines in healthy and immunocompromised participants and showed robust validation by accurately predicting neutralizing antibody kinetics in response to multiple doses of COVID-19 mRNA vaccines. Importantly, by estimating population vulnerability to breakthrough infections, we predicted tailored vaccination dosing schedules to minimize breakthrough infections, especially for immunocompromised individuals. We identified that the optimal vaccination schedules vary from CDC-recommended dosing, suggesting that the model is a valuable tool to optimize vaccine efficacy outcomes during future outbreaks.

## Graphical abstract

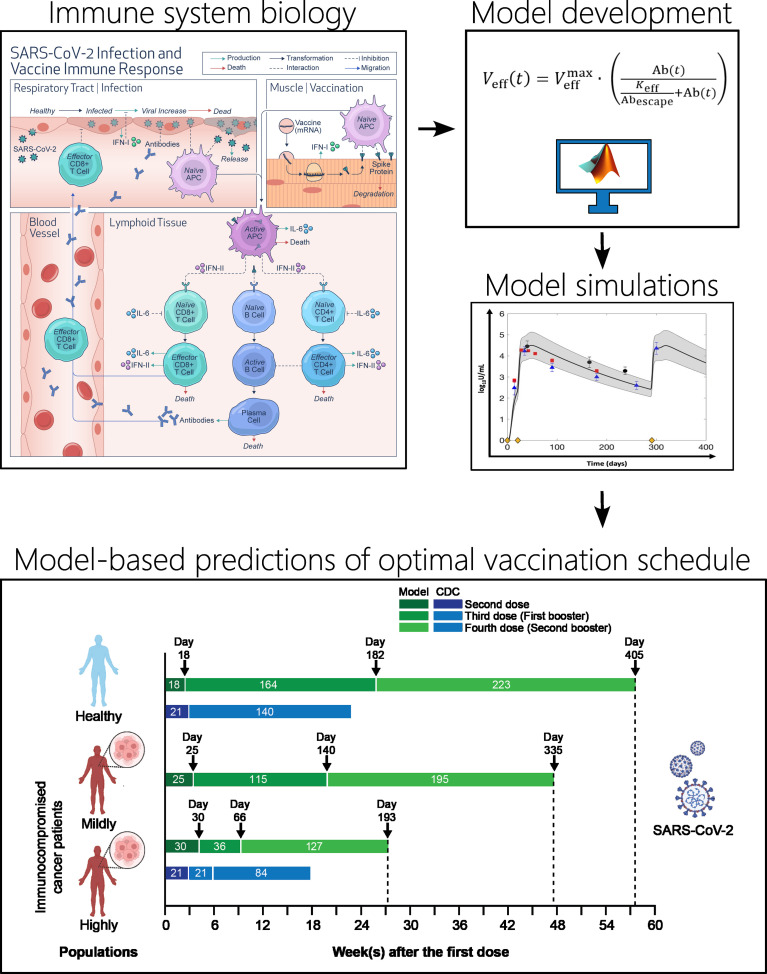

## Introduction

Since December 2019, the COVID-19 pandemic caused by SARS-CoV-2 has afflicted more than 764 million individuals and caused more than 6.91 million deaths worldwide (1). Global vaccination programs along with public health measures such as social distancing and masking are anticipated to be the most effective approaches to attain herd immunity and curb the pandemic (2, 3). Herd immunity represents a scenario where a virus cannot spread due to a dearth of susceptible hosts and can be achieved through natural infection and/or vaccination of the population. In December 2020, the first COVID-19 vaccine obtained Emergency Use Authorization from the US Food and Drug Administration, and as of December 2022, 50 vaccines have obtained regulatory approval in at least 1 country (4). As a result, over 64.3% of the world population is fully vaccinated, and about 70% of the population has received at least a single dose of a COVID-19 vaccine. However, due to the inequitable allocation of vaccines, only about 30% of the people in low-income countries have received at least a single dose (5, 6), which can facilitate the emergence of new variants of SARS-CoV-2 and thus resurgence of the pandemic.

According to a meta-analysis, seroconversion rates related to the development of neutralizing antibodies in the sera of individuals doubly vaccinated with COVID-19 vaccines are dependent on patient immunological health status; seroconversion positivity in immunocompetent individuals can be up to 99%, while in immunosuppressed patients the efficacy of vaccination varies for different diseases (e.g., solid tumors ~92%, immune-mediated inflammatory diseases ~78%, hematological cancers ~64%, and organ transplant recipients ~27%) (7, 8). Due to limited protection, immunocompromised individuals are more vulnerable to infection and are at a higher risk of developing severe or lethal COVID-19. Thus, immunizing the majority of the population is a means to additionally protect individuals who are susceptible or unable to receive a vaccine.

However, the emergence of breakthrough infections is still a major challenge. The key biological reasons for breakthrough infections are (i) waning immunity over time and (ii) emergence of mutant variants of SARS-CoV-2, referred to as variants of concern (VOCs) (9, 10). Depending on demographics and the type of vaccine administered, the humoral response (i.e., neutralizing antibodies) against SARS-CoV-2 has been found to be substantially reduced within about 6 months after 2-dose vaccination (11–13). Thus, vaccines with an initial effectiveness of 90% are only approximately 30%–70% effective after 6 months (14–16). Further, coronaviruses tend to have high genetic diversity due to their large genome size (26.4–31.7 kb), high mutation rate caused by a low-fidelity viral polymerase (~10^–4^ substitutions per site per year), and high recombination frequency (up to 25% for the entire genome in vivo) (17). As a result of selection pressure imposed by neutralizing antibodies on viral surface proteins, particularly the receptor binding domain (RBD) and the N-terminal domain (NTD) of the spike protein, which are the targets of most of the COVID-19 vaccine-induced neutralizing antibodies, SARS-CoV-2 shows clusters of mutations as documented in the genomes of VOCs (18). Mutations that confer greater fitness such as increased transmission rates and improved antibody escape are positively selected, leading to antigenic drift that makes the vaccination-induced neutralizing antibodies partially ineffective against the mutant strains (17). This predisposes vaccinated or previously infected individuals to breakthrough infections (19) (though the severity of symptoms tends to be milder) (20).

Currently, additional (booster) doses of COVID-19 vaccines are being used to reinforce protection and minimize breakthrough infections (21–24). Boosters have been administered to fully vaccinated individuals since about June 2021, except in low-income countries (25), and prioritized for high-risk populations, such as elderly and immunocompromised patients (26). According to the CDC, a 2-dose schedule (3- to 8-week gap) followed by a third dose (5-month gap) of mRNA vaccine (Pfizer-BioNTech or Moderna) is recommended for immunocompetent adults, while a 3-dose schedule (3- to 4-week gap between doses 1, 2, and 3) followed by a fourth dose (12-week gap) is recommended for immunocompromised adults (27). These scheduling recommendations were based on clinical trials performed under an unprecedented emergency scenario and generally limited to healthy volunteers, and therefore schedules may require optimization, especially for high-risk populations, to achieve better protection at the population scale.

Clinical evidence that demonstrates acceptable vaccine effectiveness, despite delayed follow-up doses, sets the premise for our theoretical investigation (28–30). Previous mathematical models that have been developed to identify optimal vaccine allocation and dosing schedules to minimize hospitalizations and deaths due to COVID-19 are primarily age-structured compartmental models, based on epidemiological principles (e.g., susceptible, exposed, infectious, and removed models), which focus on the transmission of the virus under different vaccination scenarios and the analysis of strategies to reduce the rate of infection (31–37). These models, however, lack mechanistic details relevant to the immune response to vaccines and the time-dependent variation in vaccine efficacy due to interindividual variability, vaccine efficacy against VOCs, and other biological/physiological factors. To this end, multiscale mechanistic models to study immune response to COVID-19 vaccines at the individual scale have also been developed. Voutouri et al. modeled vaccination-induced immunity to investigate the effectiveness of booster doses of COVID-19 vaccines in healthy individuals, cancer patients, and immunosuppressed patients (38). Their model considers an individual’s immune response to the initial vaccination, the timing of the booster dose, and the level of immune suppression and predicts that booster doses will be particularly beneficial for cancer patients and immunosuppressed individuals but recommends frequent dosing in these high-risk groups. Also, Korosec et al. mechanistically modeled the immune response to mRNA-based COVID-19 vaccines to study the effect of dose, age, and sex on vaccine outcomes (39). They identified a positive effect of dose on antibody titer, with older individuals exhibiting weaker response than younger and no effect of sex on vaccine-induced antibody levels. Dose dependence of immune response to adenovirus-based COVID-19 vaccines was also demonstrated in a modeling-based study by Farhang-Sardroodi et al. (40). They concluded that a delayed second dose in combination with smaller doses may be sufficient to maintain vaccine-induced protection.

Given that the above studies do not address the need to design detailed vaccination schedules for various subpopulations, as an adaptation of our previous mechanistic models of complex biological systems (41–46), we have developed a mathematical model that accurately simulates the adaptive immune response to mRNA-based COVID-19 vaccines at the individual scale and used it to optimize immune response to the vaccines at the population scale. The model was calibrated and validated with clinical data for mRNA-based COVID-19 vaccines to conduct analysis in virtual cohorts comprising immunocompetent and immunocompromised digital twins (virtual cancer patients undergoing chemotherapy and/or immunotherapy). The model identified optimal schedules for vaccination doses that minimize vulnerability to breakthrough infections, especially against VOCs (specifically Omicron), while retaining vaccine efficacy above the protection threshold in populations with different health statuses.

## Results

### Model calibration.

**Figure 1:**
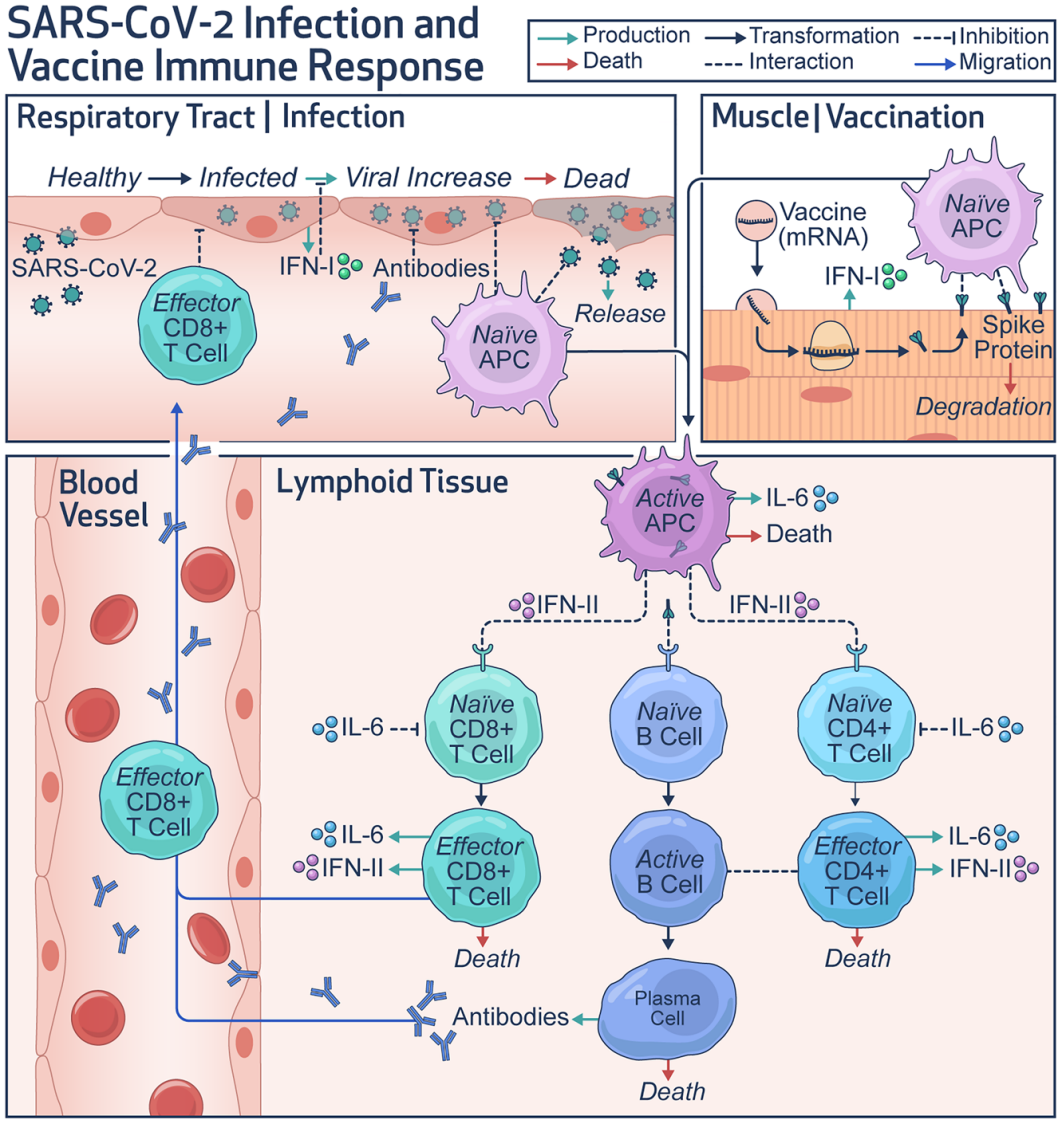
Model schematic. Diagram shows key variables and system interactions incorporated into the mathematical model. Upon respiratory tract infection by SARS-CoV-2 or intramuscular administration of mRNA vaccines, antigen-presenting cells (e.g., macrophages) engage the adaptive immune system to produce antibodies and activate T lymphocytes to build immunity against infection. Cytokines secreted by infected cells (e.g., IFN-I) and immune cells (e.g., IFN-II, IL-6) in the process have modulatory effects on the immune system. IFN-I, type I interferon; IFN-II, type II interferon; IL-6, interleukin-6.

**Figure 2:**
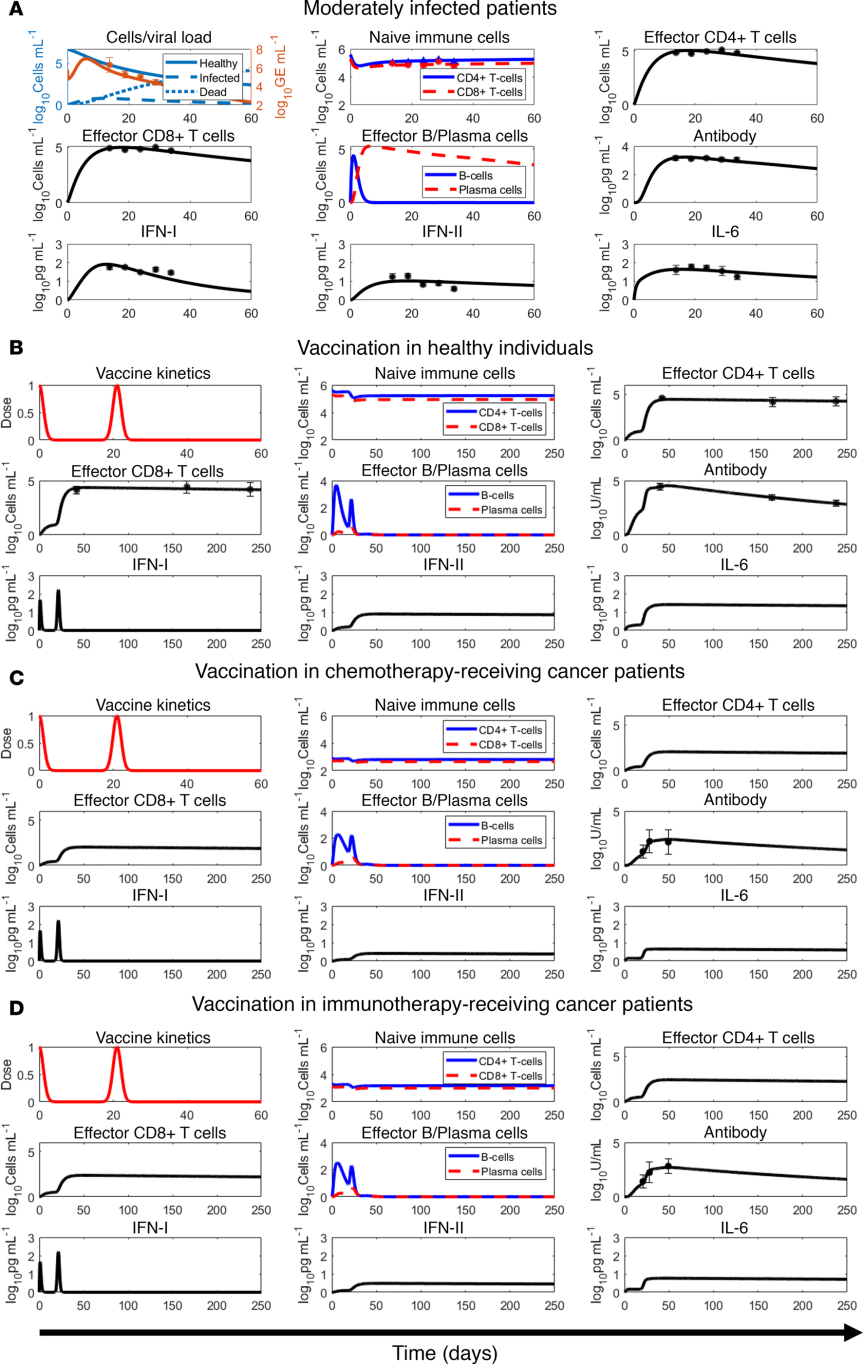
Model calibration. Model calibration with literature-derived clinical data of immune system response kinetics during (**A**) SARS-CoV-2 infection in moderately infected patients, as well as vaccination in (**B**) healthy individuals, (**C**) cancer patients receiving chemotherapy, and (**D**) cancer patients receiving immunotherapy. For consistency, all immunization data were based on 2 doses of the Pfizer-BioNTech COVID-19 mRNA vaccine. Solid or dashed lines indicate model simulations; markers with error bars represent mean ± SD values.

The focus of this work was to mechanistically model the individual-scale immune response to COVID-19 vaccines and apply it to optimize vaccine dosing schedules to maximize protection against SARS-CoV-2 and thus minimize breakthrough infections in the population (Figure 1). For this purpose, we began by fitting the model to immune response kinetics of SARS-CoV-2 infection (47), which allowed us to estimate several unknown model parameters relevant to key immune response variables that were otherwise difficult to compute from vaccination data alone (Table 1). This enabled the reliable simulation of immune response kinetics following infection. As shown in Figure 2A, the numerical solutions of the model are in agreement with the clinical data for viral load and immune response kinetics following SARS-CoV-2 infection (47). This is also indicated by the strong Pearson correlation between the observations and the model fits (Supplemental Figure 3A; supplemental material available online with this article; https://doi.org/10.1172/jci.insight.169860DS1; *R* > 0.99). The computed kinetics of viral load in the respiratory tract predict an incubation period of 8 to 9 days, which is in accordance with values established in the literature (48). Moreover, the simulations closely approximated the kinetics for 8 additional cellular and molecular immune response variables, including naive and effector lymphocytes, antibodies, interferons, and interleukins. This suggests that the model predictions are within physiological limits and thus the estimated parameter values are reliable. The results also showed that the viral load peaks around day 10, reaching a level of ~10^7^ GE/mL, while adaptive immunity variables (lymphocytes, neutralizing antibodies) peaked at around day 15, which led to clearing of the infection within 5 weeks without any pharmacological intervention.

Subsequently, as shown in Figure 2B, we calibrated the model with the clinical data obtained from healthy individuals vaccinated with mRNA vaccines (specifically Pfizer-BioNTech) (49). For this purpose, a double dose of the vaccine was simulated in accordance with the schedule used for the individuals in the study (49). A Gaussian function described the kinetics of antigen load following injections on days 0 and 28 (Supplemental Methods, Equation S1). The solutions for the various immune response variables were computed over a period of 8 months and fitted to the available clinical data for effector T cells (CD4^+^ and CD8^+^) and neutralizing antibodies. Our results showed a high degree of correlation between the model fits and clinical measurements (Supplemental Figure 3B; *R* > 0.96). To ensure that the model can reproduce immune response elicited by the vaccines over long periods, some of the parameters were refitted (Table 1). Since during the previous calibration (i.e., infection), the characteristic time of simulation was a few weeks unlike the vaccination scenario where the simulated time is a few months, we recalibrated some parameters to ensure long-term accuracy of the model. Also, to capture any fundamental differences between immune response to infection and vaccines (50, 51), or to account for variation in units of measurement between experiments, we performed recalibration of model parameters linked to antigen presentation, lymphocyte death, and antibody production and clearance. An important observation is the gradually waning levels of neutralizing antibodies and effector lymphocytes, which suggests that protection conferred by mRNA vaccines is temporal, warranting the use of boosters.

To accurately represent the vaccine-induced immune response in immunocompromised individuals, we also calibrated the model with clinical data obtained from vaccinated cancer patients undergoing chemotherapy or immunotherapy (Figure 2, C and D) (52). In both cases, we assumed that due to the underlying pathophysiology and associated treatment, the levels of some immune system parameters were only a fraction (0 < *f* < 1) of their values in healthy individuals (*f* = 1). Therefore, keeping all other model parameters from the previous 2 fits as constants, we fitted the model to 2 data sets (52) to estimate the parameter *f*, which resulted in a value of *f* = 0.517 for chemotherapy-treated patients and *f* = 0.588 for immunotherapy-treated cancer patients. The model fits were also in good agreement with clinical data (Supplemental Figure 3, C and D; *R* > 0.96).

### Model validation.

**Figure 3:**
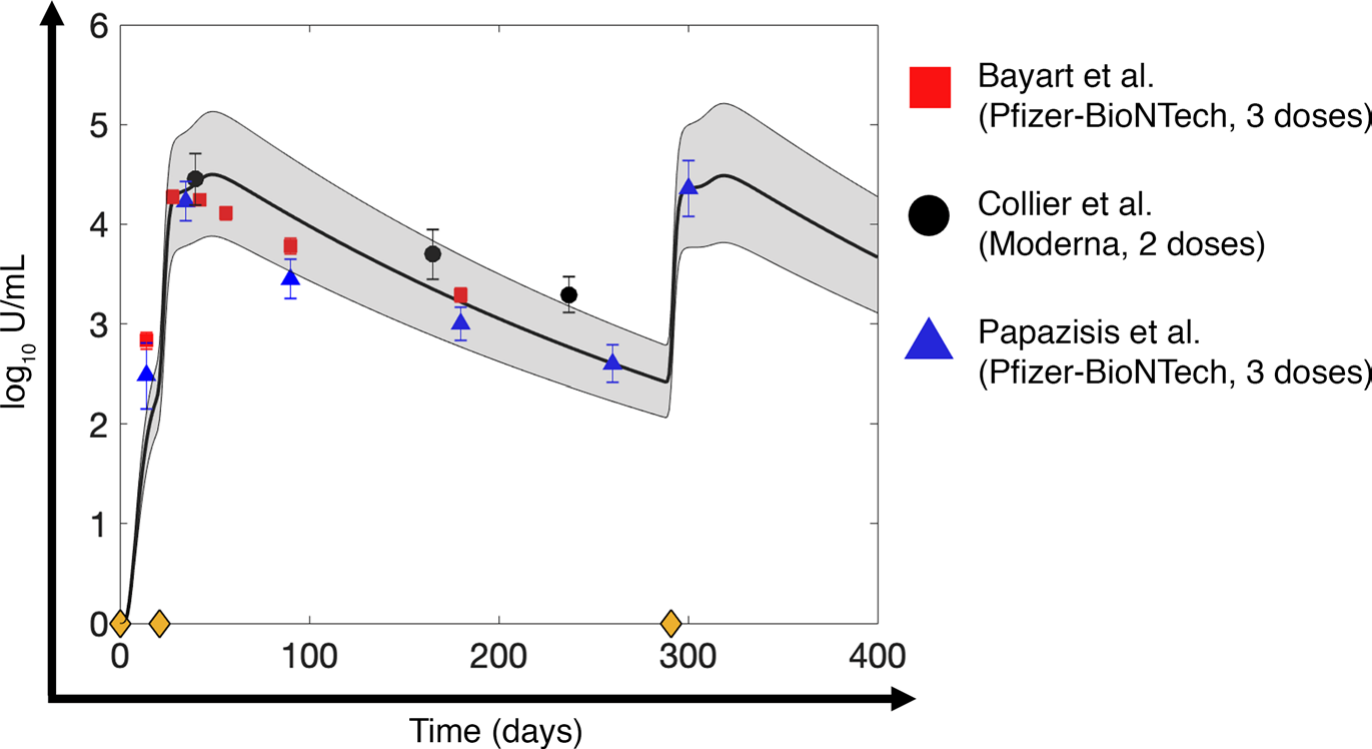
Model validation. Validation of the mathematical model with antibody kinetics data derived from the literature for healthy individuals vaccinated with 2 doses of Pfizer-BioNTech COVID-19 mRNA vaccine (red squares) (53), 2 doses of Moderna COVID-19 mRNA vaccine (black circles) (49), and 3 doses of Pfizer-BioNTech COVID-19 mRNA vaccine (blue triangles) (54). Solid line indicates model predictions, gray bands represent 90% prediction intervals, and markers with error bars represent mean ± SD values of clinical data. Yellow diamonds on the *x* axis denote timing of injection (i.e., first dose given on day 0, second dose given on day 21 after first dose, and third dose given 9 months after second dose).

To test the ability of our model to accurately reproduce the immune response to mRNA vaccines, we simulated 2 and 3 doses of the Pfizer-BioNTech and Moderna COVID-19 vaccines in healthy individuals (data not used for calibration). As shown in Figure 3 and Supplemental Figure 4 (*R* = 0.92), the computed neutralizing antibody (IgG) kinetics closely resembled the literature-derived clinical data following 2 doses of the Pfizer-BioNTech COVID-19 vaccine (53), 2 doses of the Moderna COVID-19 vaccine (49), and 3 doses of the Pfizer-BioNTech COVID-19 vaccine (54). The dosing schedules were obtained from the respective clinical studies, and the parameter values were based on the values calibrated for healthy individuals in the previous section (Table 1). The ability of the model to accurately predict the response to the third dose, despite not using the third dose data during model calibration, highlights the biological and physiological robustness of our mechanistic model. Having established the validity of our model to reliably reproduce neutralizing antibody kinetics with various mRNA vaccines and dosing schedules, we proceeded to perform numerical experiments to explore the heterogeneity in immune responses and optimize dosing schedules to minimize breakthrough infections.

### Sensitivity analysis.

**Figure 4:**
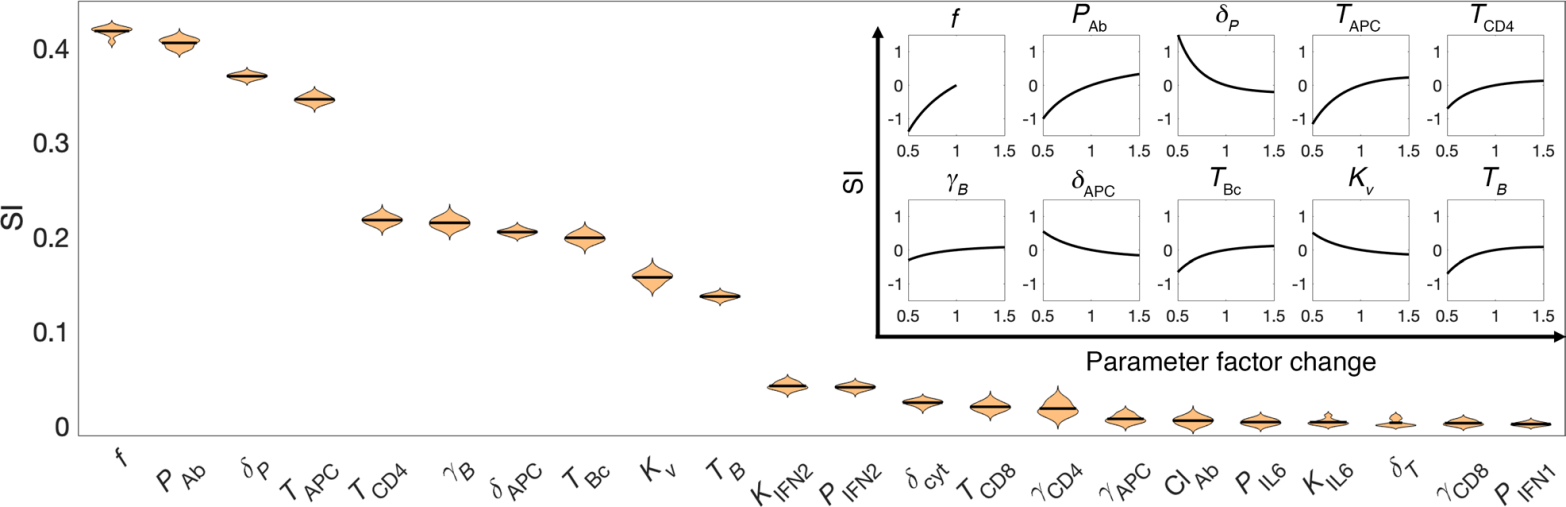
Parameter sensitivity analyses. Violin plot showing parameters ranked in descending order (from left to right) for their sensitivity, as indicated by the sensitivity index (SI) obtained through GSA. Correlation of parameter perturbation and its effect on antibody titer (quantified as SI) obtained through LSA for the top 10 ranking parameters of GSA is shown in the inset. Note that parameters were perturbed in the range of ±50% around their baseline values for both GSA and LSA.

To study the relative significance of model parameters in governing humoral response to mRNA-based COVID-19 vaccines, quantified as AUC of neutralizing antibody titer kinetics following a unit dose of the vaccine, global sensitivity analysis (GSA) and local sensitivity analysis (LSA) were performed with 22 model parameters that characterize the key immunological interactions, processes, and immune states considered important for vaccine-induced immune response generation. As shown in Figure 4 and Supplemental Figure 5, the immunosuppression factor *f* stands out as the most relevant parameter in determining antibody response to vaccines, which highlights the importance of immune health status (i.e., immune cell sufficiency) in governing vaccine-induced protection. Further, as shown in the inset of Figure 4, LSA reveals a positive monotonic correlation between change in *f* and SI for antibody titer, which signifies better antibody response in immunologically competent individuals and thus warrants the need for additional doses or optimized dosing frequency in immunocompromised patients (8). Following this, biological parameters that characterize antibody production *P*_Ab_, death of antibody secreting plasma cells *δ_P_*, and antigen-induced activation of naive APCs *T*_APC_ were observed to also influence antibody response strongly (Figure 4), thereby indicating the relevance of antigen presentation, plasma cell population, and antibody secretion from plasma cells in humoral immunity development. While the relationship between change in antibody titer and perturbations of parameters *P*_Ab_ or *T*_APC_ was monotonically increasing within the studied parameter range, that between antibody titer and *δ_P_* was monotonically decreasing (Figure 4, inset). Further, additional parameters belonging to CD4^+^ T cell activation *T*_CD4_, growth of B cells *γ_B_*, death of activated APCs *δ*_APC_, differentiation of B cells into antibody-secreting plasma cells *T*_BC_, potency of the injected antigen to activate naive APCs *K_v_*, and activation of naive B cells *T_B_* were also observed to have a moderate effect on antibody titer (Figure 4). This further validates the relevance of previously identified processes, in addition to the levels of activated CD4^+^ T cells and B cells in governing antibody response. While the parameters identified in this analysis do not contribute directly to immune health status in our model, except *f*, their significance in antibody response warrants their inclusion in virtual patient cohort generation to study the effect of the underlying population-scale biological variability on vaccine-induced protection.

### Heterogeneity in immune response to vaccines at the individual and population scale.

To study the influence of (i) vaccine dosing schedules and (ii) the immune status of an individual on neutralizing antibody levels and vaccine efficacy, we simulated immune responses under different dosing schedules in representative healthy and immunocompromised patients. Based on the dosing schedules used across various countries, we considered 3 vaccination regimens: *rapid*, *intermediate*, and *delayed*. In all cases the first dose was given on day 0; (i) rapid: second dose is given 2 weeks after the first dose, and the first booster (third dose) is given 5 months after the second dose; (ii) intermediate: second dose is given 4 weeks after the first dose, and the first booster is given 7 months after the second dose; (iii) delayed: second dose is given 8 weeks after the first dose, and the first booster (third dose) is given 9 months after the second dose.

Here, the immune health status was defined by the nondimensional, empirical parameter *f*, such that healthy individuals have *f* = 1, mildly immunocompromised patients have *f* = 0.75, and highly immunocompromised individuals have *f* = 0.55. As previously discussed, *f* = 0.55 corresponds to cancer patients undergoing chemotherapy or immunotherapy, whereas *f* = 0.75 simulates individuals with underlying conditions that may also affect the immune system but usually to a lesser degree (e.g., autoimmune diseases). As per evidence in the literature, plasma antibody titer is a correlate of protection against infection (14, 55). Therefore, we used the computed neutralizing antibody levels as predictors of vaccine efficacy (i.e., protection against SARS-CoV-2; see Methods).

**Figure 5:**
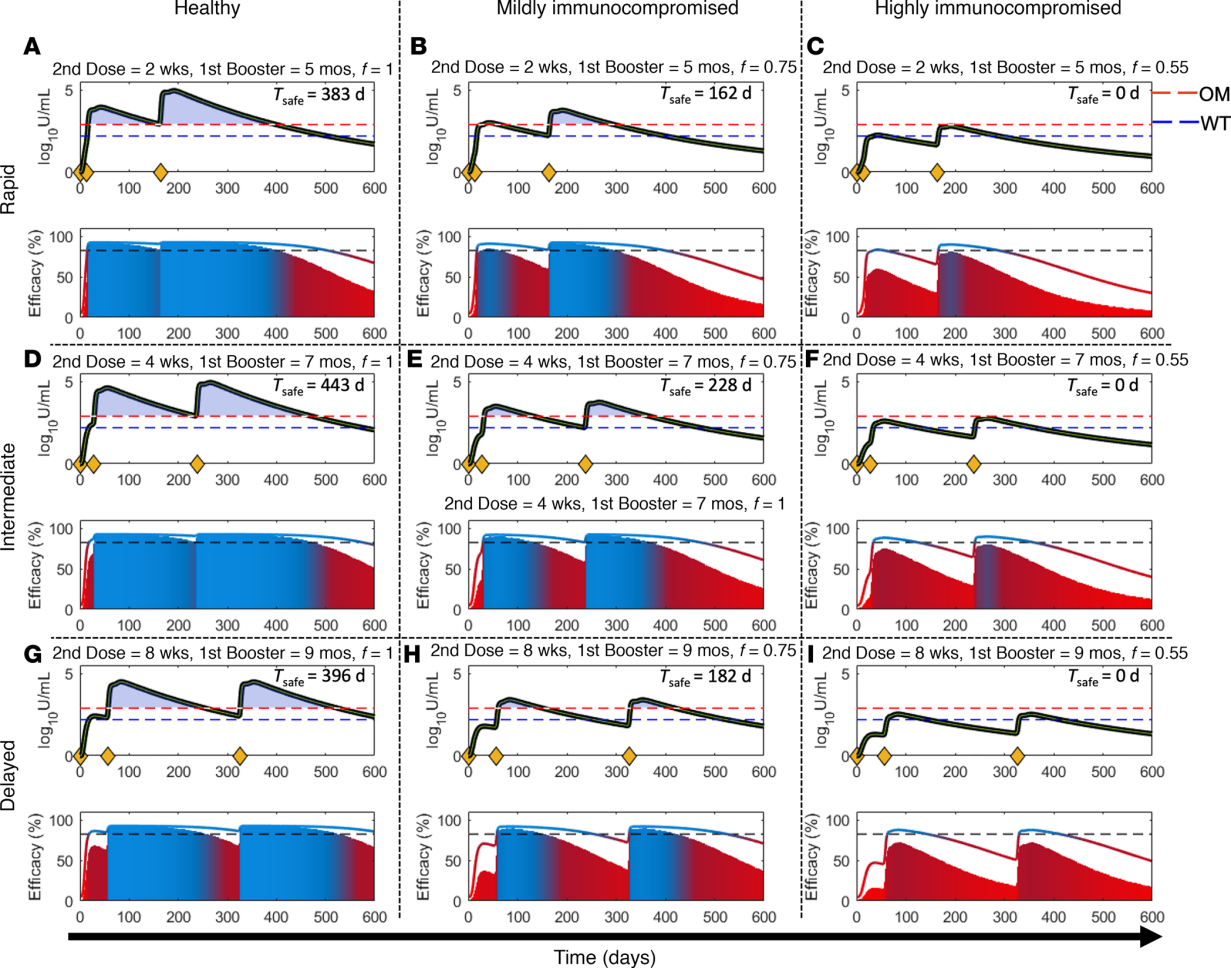
Effect of vaccine dosing schedule and immune health status on antibody levels and vaccine efficacy. Simulations in representative (**A**, **D**, and **G**) healthy and (**B**, **C**, **E**, **F**, **H**, and **I**) immunocompromised individuals show antibody levels and vaccine efficacy against wild-type strain (WT) and Omicron variant (OM) of SARS-CoV-2 following (**A**–**C**) rapid, (**D**–**F**) intermediate, and (**G**–**I**) delayed vaccine dosing schedules. Yellow diamonds on the *x* axes, in each upper subpanel, indicate injection time points. In each upper subpanel, the black solid line represents antibody levels, with the dashed blue and red lines indicating protective threshold against WT and OM, respectively. The lower subpanel shows vaccine efficacy (colored solid line for WT and shaded area for OM), with the dashed black line indicating the 82.3% threshold of protection. Note: The value *T*_safe_ indicated in every upper subpanel represents the number of days when antibody levels are above the protective threshold for both WT and OM.

We used the model to predict the humoral response to mRNA vaccines following 3 dosing schedules in representative healthy or immunocompromised individuals for a 600-day period. As shown in Figure 5, A, D, and G (upper subplot in each panel), irrespective of the dosing schedule, the antibody levels remained above the protection threshold for both OM (770 U/mL) and WT strain (154 U/mL) for a much longer duration in healthy individuals (as indicated by the shaded gray area and quantified as the *T*_safe_ value = 383–443 days) than in mildly immunocompromised individuals (Figure 5, B, E, and H; *T*_safe_ 162–228 days). In contrast, in cancer patients undergoing antineoplastic treatment (i.e., highly immunocompromised participants), *T*_safe_ was 0 days across all dosing schedules (Figure 5, C, F, and I). This suggests that highly immunocompromised individuals are vulnerable to infection with OM throughout the 600-day simulation period; however, protection against WT is intermittently present depending upon the dosing schedule. Of note, within both the healthy and mildly immunocompromised individuals, the intermediate dosing schedule led to higher *T*_safe_ values (443 days if healthy, 228 days if mildly immunocompromised; Figure 5, D and E) than the rapid dosing schedule (383 days if healthy, 162 days if mildly immunocompromised; Figure 5, A and B) or the delayed dosing schedule (396 days if healthy, 182 days if mildly immunocompromised; Figure 5, G and H). Nonetheless, the protection window in these cases is not continuous for the chosen dosing schedules, and an intermediate “gap” is observed between the second dose and first booster (third dose) that highlights the period when antibody levels temporarily fall below the protective threshold for OM and/or WT. The duration of this gap varies according to immune health status and dosing schedule.

The corresponding vaccine efficacy kinetics are shown in the lower subplots in Figure 5. The shaded area represents the vaccine efficacy against OM, and the solid-colored line indicates the vaccine efficacy against WT. The continuous color mapping assigns blue to efficacies above the protection threshold (>82.3%) and red to efficacies equal to or below the protection threshold (≤82.3%). As visible from the bluish region of the shaded area, for any given dosing schedule, healthy individuals have greater vaccine efficacy against OM than immunocompromised individuals. In highly immunocompromised individuals the shaded area always remained below the protective threshold (82.3%), indicating a high risk of becoming infected with OM (Figure 5, C, F, and I). As expected, due to limited *antibody escape* (56), the vaccine efficacy against WT was greater than that against OM in all individuals under all dosing schedules (as indicated by the colored solid line). Further, in healthy individuals, the 3 dosing schedules produced antibody titers above the WT protection threshold for the majority of the simulation period (Figure 5, A, D, and G). In mildly immunocompromised individuals, protection against WT did not persist continuously (Figure 5, B, E, and H). For example, in the delayed dosing schedule shown in Figure 5H, the period between day 236 and day 330 (~3 months) indicates a vaccine efficacy of less than 82.3%. In highly immunocompromised cases, the 3 dosing schedules provided limited protection against WT, with prolonged periods of lapse in immunity. Though we only considered representative individuals, these observations collectively highlight the importance of optimizing the dosing schedule based on the immune health status of a subpopulation to achieve continuous, long-term protection against both WT and other VOCs (e.g., OM).

**Figure 6:**
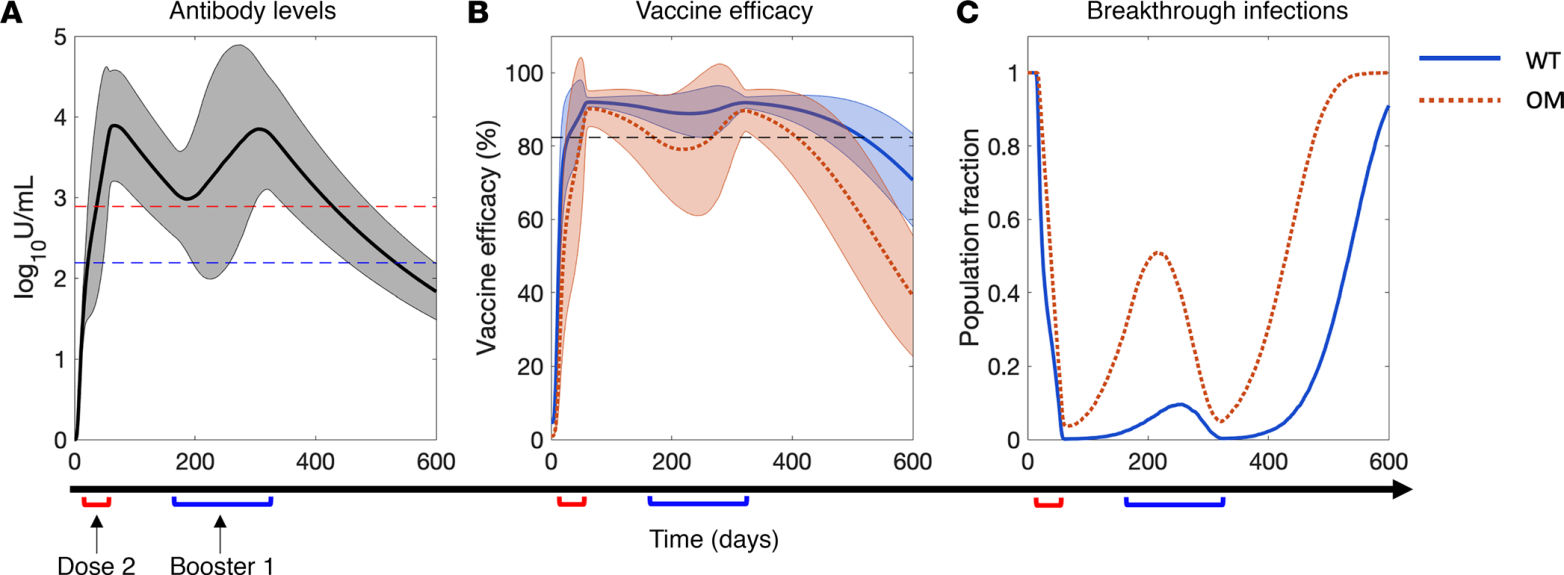
Effect of heterogeneity in vaccine dosing schedules and immune health status on breakthrough infections at the population scale. (**A**) Average antibody levels in plasma, (**B**) corresponding vaccine or antibody efficacy, and (**C**) population fraction vulnerable to breakthrough infections due to wild-type strain (WT, solid blue line) and Omicron variant (OM, dotted orange line) of SARS-CoV-2 over time. Solid and dotted lines in **A** and **B** represent average behavior of 10,000 simulated individuals, and shaded bands indicate 90% prediction interval. Note that the first dose was administered on day 0 to each simulated individual, the second dose was administered between day 14 and day 56, and the third dose (i.e., first booster) was administered between day 150 and day 270. Red and blue brackets on *x* axis denote timing windows with respect to day 0 for second dose and third dose, respectively, used to design unique vaccine schedules in model simulations. Immune health status (*f*) of the simulated population varied between 0.5 and 1.

To evaluate the effects of dosing schedules and immune health status on the variability in immune response to mRNA vaccines at the population level, we simulated the vaccination of a virtual population of 10,000 individuals with 3 doses (cohort A; see Methods for details of dosing schedule) and assessed the corresponding vulnerability to breakthrough infections. Note that the dosing schedule for each simulated individual was obtained randomly from continuous time intervals (red and blue brackets on *x* axis of Figure 6) to replicate the real-world heterogeneity in dosing time intervals. As shown in Figure 6A, the average antibody kinetics across the 10,000 individuals remained above the protective threshold for OM and WT. However, for a substantial fraction of the population, antibody levels remained below the OM threshold for a prolonged period (~5 months). This is evident from the shaded area representing 90% prediction interval. Further, translating the antibody levels to vaccine efficacy using Equation 2, we observed that for a significant fraction of the 10,000 individuals, vaccine efficacy against OM fell below the 82.3% protection threshold (see Figure 6B, orange shaded area). Subsequently, we quantified the fraction of the virtual population that presented a vaccine efficacy below the protective threshold for OM and WT (Figure 6C, see Methods). This population fraction can alternatively be interpreted as the fraction of vaccinated individuals in a population that is vulnerable to breakthrough infections, i.e., becoming infected despite being vaccinated. As observed in Figure 6C, this fraction increased to about 0.5 (or ~50% of the population) for OM in vaccinated individuals (2 doses), then declined rapidly following administration of the first booster (third dose). However, due to waning antibody levels, which translated into declining efficacy, the vulnerable fraction began to increase again and became 1 (i.e., 100% of population) in about 6 months after the booster window. In contrast, for WT, the vulnerable fraction of the population peaked at about 0.1 (or ~10% of the population) in vaccinated individuals (2 doses) and then decreased again after administration of the first booster (third dose), suggesting effective protection against WT in vaccinated individuals for up to approximately 1.5 years, irrespective of the dosing schedule or immune health status. Of note, in the population-scale simulation, immune health status was nonuniformly distributed across the population, as defined by the left half-Gaussian distribution (Supplemental Figure 2C); this indicates that a major proportion of the population is healthy. It is worth mentioning that the sharp rise in Figure 6C of the population fraction several months after the first booster warrants the administration of a second booster to curb the vulnerability to VOCs and WT. Given that the proposed dosing schedules do not warrant continuous protection against VOCs and/or WT, it is imperative to optimize the schedules to achieve long-term protection in the population without lapses.

### Vaccine dosing schedule optimization.

Following the previous numerical experiments, we intended to identify optimal vaccine dosing schedules to achieve continuous protection against OM (as a representative example) for prolonged periods. We generated 3 virtual cohorts of 10,000 individuals (cohort B) each to represent healthy, mildly immunocompromised, and highly immunocompromised individuals, then implemented several dosing schedules to identify optimal times for the second dose, the third dose (first booster), and the fourth dose (second booster) in each subcohort (see Methods).

**Figure 7:**
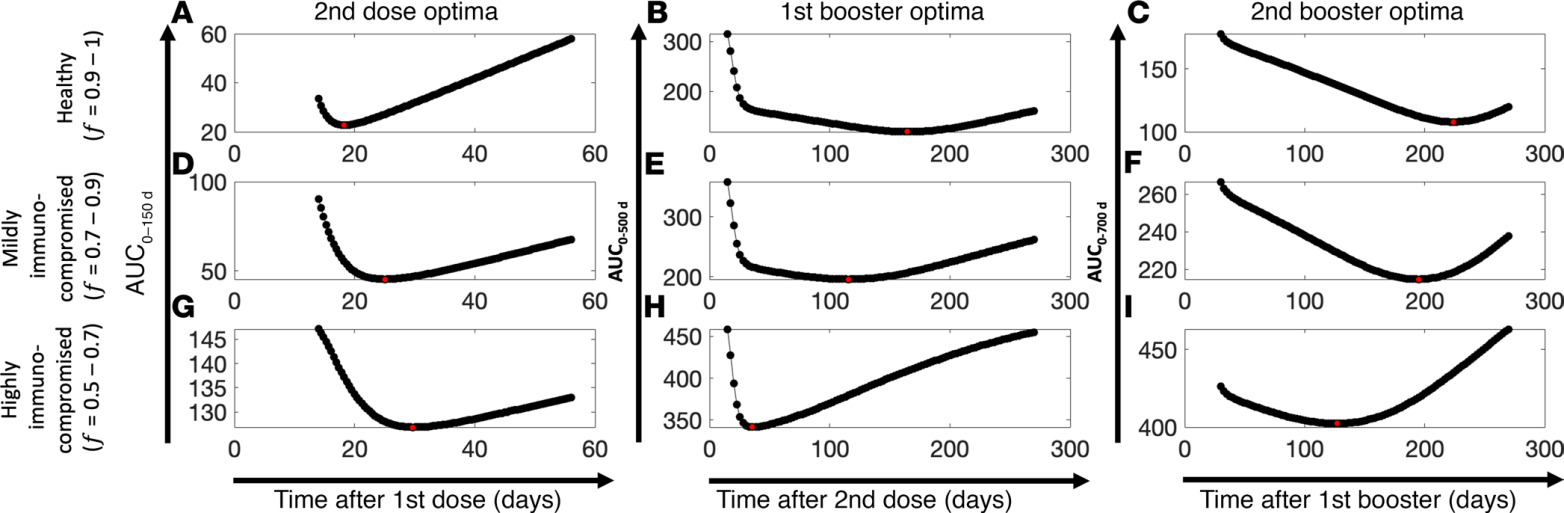
COVID-19 vaccine dosing schedule optimization. Area under the curve (AUC) of breakthrough infection vulnerability kinetics curve obtained from simulation of 10,000 individuals from different population subtypes under unique dosing schedules and immune health status. Estimated AUC versus dosing schedules for (**A**, **D**, and **G**) dose 2, (**B**, **E**, and **H**) booster 1 (i.e., dose 3), and (**C**, **F**, and **I**) booster 2 (i.e., dose 4) for (**A**–**C**) healthy, (**D**–**F**) mildly immunocompromised, and (**G**–**I**) highly immunocompromised individuals, obtained through model simulations. Each black dot represents 1 AUC value. Red dot in each plot represents the corresponding minima for each dose and population subtype.

As shown in Figure 7, the AUC of vulnerability kinetics curves followed a nonlinear relationship with respect to dosing schedules, and a minimum is visible for each dose and population subtype (highlighted by a red circle). As shown in Figure 7, A, D, and G, as the immune status changed from healthy to highly immunocompromised, the position of the minima on the *x* axis showed a right shift, such that the optimal time for the second dose in healthy, mildly immunocompromised, and highly immunocompromised individuals was 18, 25, and 30 days after the first dose, respectively. In contrast, as shown in Figure 7, B, E, and H, the minima for the first booster showed a left shift on the *x* axis from healthy to highly immunocompromised individuals, such that the optimal time for first booster was 164 days (~5.5 months), 115 days (~4 months), and 36 days (1.2 months) after the second dose for healthy, mildly immunocompromised, and highly immunocompromised individuals, respectively. Similarly, as shown in Figure 7, C, F, and I, the minima for the second booster showed a left shift from healthy to highly immunocompromised individuals, such that the optimal schedule for the second booster was 223 days (~7.5 months), 195 days (~6.5 months), and 126 days (~4 months) after the first booster for healthy, mildly immunocompromised, and highly immunocompromised individuals, respectively.

It is intuitive to expect intervaccination periods to be longer for healthy individuals than for immunocompromised patients. This is evidenced by data presented in Figure 5, where the antibody level stayed above the OM protection threshold for a longer period in healthy individuals than in their immunocompromised counterparts, thereby allowing the possibility to delay subsequent doses. Although this is true for the first and the second boosters (Figure 7B vs. Figure 7, E and H; Figure 7C vs. Figure 7, F and I), the trend is reversed for the second dose (Figure 7A vs. Figure 7, D and G), where healthy individuals seem to require the second dose sooner than immunocompromised individuals to ensure continuity of protection against OM. This observation can be explained considering a key mechanistic assumption of our model. Recall that the immune health status parameter *f* scales the homeostasis level of naive immune cells (CD4, CD8, B). In immunocompromised individuals, *f* ranged from 0.5 to 0.9; therefore, the homeostasis level of naive immune cells was less than that in healthy individuals (Figure 2, B–D). As a result, when the second dose was given too soon after the first dose in immunocompromised individuals, due to reduced levels of CD4^+^ T cells and therefore slower activation of B cells, the production of neutralizing antibodies from plasma cells could be thwarted, thereby rendering an individual vulnerable to infection. Therefore, permitting the CD4^+^ T cell and B cell population to regenerate after the first dose will allow antibody titers to rise to levels associated with adequate protection. Of note, since healthy individuals produce or activate immune cells more quickly (given *f* = 1), they are ready to receive a second dose sooner than immunocompromised individuals. However, in the case of healthy individuals, as shown in Figure 7A, the AUC_0–150 d_ values were smaller than those of immunocompromised patients (Figure 7, D and G) for up to ~6 weeks of delay after the first dose. A 6-week delay after the first dose predisposed ~30% (obtained from the ratio of AUC_0–150 d_ value at 6 weeks, i.e., ~45, to maximum possible value of AUC_0–150 d_, i.e., 150) of the healthy population to a breakthrough infection over 150 days under no public health restrictions. This indicates that although an optimal waiting period for healthy individuals is two-and-a-half weeks after the first dose (which predisposes only ~15% of the healthy population over 150 days), if required due to logistic constraints, waiting longer (up to 6 weeks) will still allow the healthy individuals to be more protected than immunocompromised individuals.

### Testing model-predicted optimal dosing schedules.

**Figure 8:**
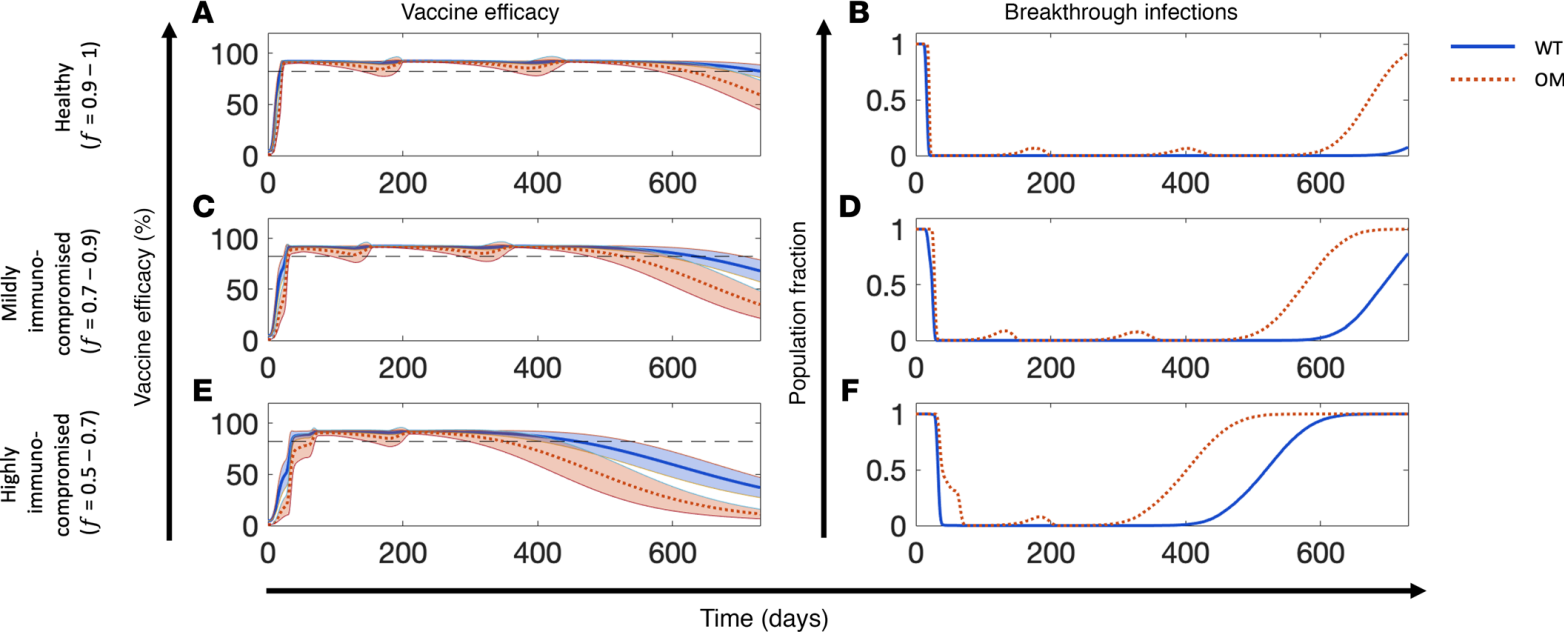
Testing model-predicted optimal dosing schedules. (**A**, **C**, and **E**) Vaccine efficacy and (**B**, **D**, and **F**) vulnerability to breakthrough infections due to wild-type strain (WT, solid blue line) and Omicron variant (OM, dotted orange line) of SARS-CoV-2 in (**A** and **B**) healthy, (**C** and **D**) mildly immunocompromised, and (**E** and **F**) highly immunocompromised individuals. For each population subtype, testing was done on 10,000 simulated individuals with unique *f* and dosing schedule values. Colored bands represent 90% prediction intervals.

Finally, to demonstrate the impact of the previously identified optimal dosing schedules (for the second dose and the 2 boosters) in reducing vulnerability to breakthrough infections, we simulated a vaccination regimen with 4 doses in 10,000 virtual individuals per group, belonging to the 3 cohorts of interest (healthy, mildly immunocompromised, and highly immunocompromised; see cohort B in Methods), then measured the vaccine efficacy and corresponding level of vulnerability to infection over a period of 2 years. Note that to simulate a more realistic test scenario and add variability to the optimal dosing schedules identified previously (Figure 7), we sampled the dosing schedules from within a ±10 % uniform distribution around the optimal values. As shown in Figure 8, A, C, and E, the average vaccine efficacy for WT and OM was above the protection threshold in all subpopulations for an extended period, the duration of which was dependent on the viral strain and population subtype. Therefore, the corresponding vulnerability to breakthrough infections for OM and WT remained at almost 0 for most of the 2-year period in healthy individuals and showed only 2 intermittent windows of ~2 months each where the vulnerability was as high as ~0.065 (Figure 8B). In mildly immunocompromised individuals, the optimized protocol exhibited similar results, although the vulnerability to infection after the fourth dose began to rise sooner in comparison with the healthy population (Figure 8D). Furthermore, as shown in Figure 8F, in the highly immunocompromised cohort, the same trend continued; although complete protection against OM and WT was observed for a shorter duration, the results were nonetheless notably more promising compared with the observed findings in Figure 5, C, F, and I, where vaccine efficacy remained below the OM protection threshold throughout the 600-day window under conventional dosing schedules.

**Figure 9:**
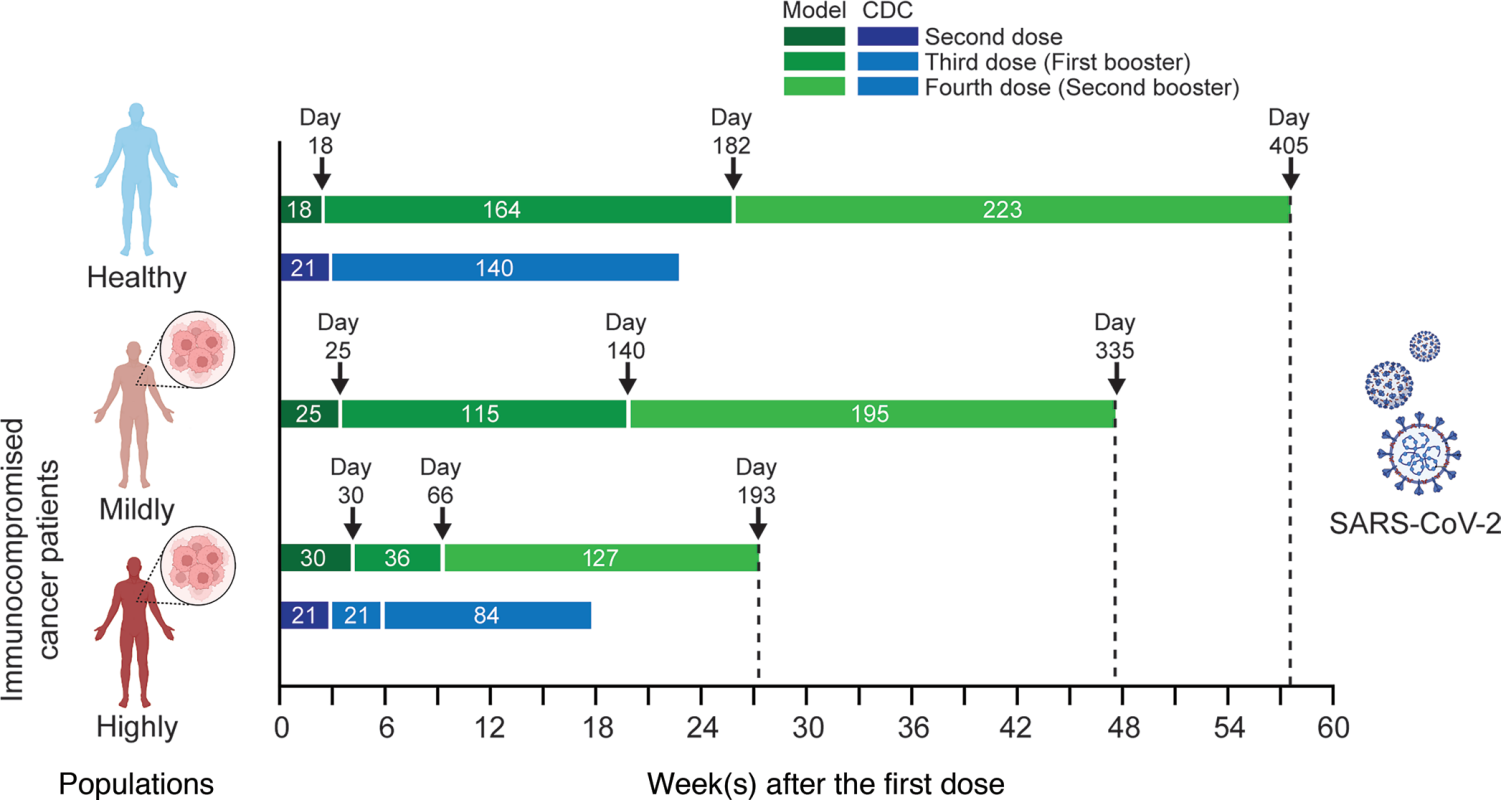
Model-predicted optimal dosing and CDC-recommended dosing schedules for the Pfizer-BioNTech vaccine in healthy and immunocompromised populations. The ongoing CDC guidelines for dosing schedules are represented by the blue bands, and those predicted by the model are shown in green.

Finally, the optimal dosing schedules identified above are summarized in Figure 9 (green bands), with a comparison made to the CDC-recommended dosing schedules being currently implemented for the Pfizer-BioNTech vaccine (blue bands). The ongoing CDC guidelines for COVID-19 vaccination for healthy individuals (not moderately or severely immunocompromised and <50 years of age) include 3 doses with intervals of 3–8 weeks between the first and second dose (represented as 21 days) and 5 months between the second and third dose (represented as 140 days). The model-predicted schedule closely recapitulates the CDC guidelines with the inclusion of a fourth dose to prolong immunity for 385 days (>1 year). Although the model distinguishes between 2 immunocompromised cancer populations (mildly and highly), the CDC guidelines suggest a schedule of 4 doses for patients who are moderately or severely immunocompromised (with intervals of 21, 21, and 84 days, respectively). According to the model-predicted optimal dosing schedule, longer gaps between doses (or boosters) would not compromise the immunity of healthy and immunocompromised patients that could represent a solution to logistic constraints.

## Discussion

We developed a mechanistic model of adaptive immune response to COVID-19 vaccines and viral infection in healthy and immunocompromised individuals. Using model-based simulations, we identified optimal vaccine dosing schedules of mRNA-based COVID-19 vaccines for immunocompetent and immunocompromised individuals to minimize breakthrough infections at the population scale. The model was formulated as a system of ordinary differential equations (ODEs) to account for key biological processes and interactions leading to the development of antigen-induced humoral and cellular immunity (Figure 1). Following calibration and validation of the model with published clinical data (Figures 2 and 3), simulations were performed to study the effects of immune health status and vaccine dosing schedules on plasma antibody titers (a correlate of protection against infection) and vaccine efficacy (Figure 5). Sensitivity analysis identified the significance of immunosuppression factor *f* in determining antibody response to vaccines, thereby highlighting the importance of immune health status (i.e., immune cell sufficiency) in governing vaccine-induced protection, in addition to parameters representing key immunological processes (Figure 4). Using these parameters, virtual cohorts were generated through Latin hypercube sampling (LHS) to characterize the effect of interindividual differences in immunity and variability in vaccine dosing schedules on the vulnerability to breakthrough infections at the population scale (Figure 6). Through immune response simulations of virtual cohorts, the model was then applied to identify optimal dosing schedules of the vaccines to minimize breakthrough infections in various cohorts (Figure 7). Through our analysis, we highlighted critical waiting windows for immunocompromised individuals (25 and 30 days after first dose for mildly and highly immunocompromised individuals, respectively) to ensure sufficient time for the development of immune recall responses and minimize vulnerability to breakthrough infections in their subpopulations. In the case of healthy individuals, while the optimal waiting period after first dose was found to be two-and-a-half weeks, we proposed that it can be extended (without much compromise to protection) up to 6 weeks. Thereby, we make the case for longer waiting period between doses without compromising the immunity at the population scale (Figure 8).

The presented model is based on generalized adaptive immune response to antigens and can thus be adapted to investigate different infections or vaccines, given appropriate data for model calibration. Through our proof-of-concept study, we have thus developed a potentially novel approach to optimize vaccine dosing schedules in case of future outbreaks. Given that the model is based on several parameters whose values are not known a priori, and as shown through a detailed GSA, multiple parameters are critical in determining humoral immune response to vaccines, which indicates that based on variation in model parameter values, the predictions of the model may vary. However, through a robust calibration and validation exercise based on multiple clinical studies, we have tried to minimize such a possibility. Through the generation of virtual patient populations based on parameter perturbation and sampling, we aimed to capture the variability in the underlying biology to predict the variation in immune response and identify strategies to maintain protection. Our approach to vaccine scheduling optimization is solely based on maintenance of antibody titer above a literature-derived threshold of protection and does not involve the epidemiological, social, or behavioral aspects associated with the transmission dynamics of infection in a population. Also, the mechanistic underpinnings of immunosuppression and innate immune response need to be considered in greater detail in future studies. The model adaptations relevant to the other types of COVID-19 vaccines will need to be considered as well. Importantly, our results also suggest the need for follow-up boosters (more frequently for immunocompromised individuals due to rapidly waning immunity) to ensure continued immunity against breakthrough infections and reinfections, especially given the emergence of novel VOCs.

Several aspects of these findings merit further comments. A mathematical modeling approach, which is data driven and based on principles of physiology, immunology, and biophysics, can be a valuable tool to simulate population-scale heterogeneity in immune health status and immune response to vaccines, thereby supporting rational design of dosing schedules. In addition, given the disparities in global vaccine allocation, optimization of dosing schedule to extend the gaps between doses with no major effect on efficacy could allow for improved distribution of vaccines to countries without the capacity to provide for themselves, reduce costs, and promote vaccine compliance, thereby benefiting the overall population, but especially patients in critical care.

## Methods

### Model development.

Based on our previous mathematical modeling of the immune response to SARS-CoV-2 infection (57), we developed a model of the adaptive immune response to COVID-19 vaccines. As shown in Figure 1, the model incorporates key biological processes that are relevant to antigen presentation at the site of vaccination (i.e., muscle), the development of adaptive immune responses in the lymphoid tissue, and protection against infection in the respiratory tract. The model was formulated as a system of ODEs (Supplemental Methods, Equations 1–17), which describe the kinetics of key immune response variables following vaccination or infection. The equations were solved numerically as an initial value problem in MATLAB R2018a. While some of the model parameters were known a priori (Table 1), the remainder were estimated by nonlinear least squares fitting of the model to multiple clinical data sets obtained from the literature (47, 49, 52). The model was then used to simulate the immune response to mRNA-based COVID-19 vaccines in healthy and immunocompromised populations and was implemented to identify optimal vaccine dosing schedules to minimize breakthrough infections. The model equations are described in detail in Supplemental Methods.

### Model calibration and validation.

Using the built-in MATLAB function *lsqcurvefit*, nonlinear least squares regression was performed to fit the model to literature-derived clinical data to estimate the unknown model parameters (Table 1). The data sets used for model calibration included: (i) viral load and immune response kinetics following a SARS-CoV-2 infection (47), (ii) immune response kinetics following vaccination with mRNA vaccines in healthy individuals (49), and (iii) cancer patients undergoing chemotherapy or immunotherapy (52). Further, to test the predictive ability of our model to accurately reproduce the immune response to mRNA vaccines, we simulated 2 and 3 doses of the Pfizer-BioNTech and Moderna vaccines in healthy individuals using the parameters obtained from model calibration for the healthy population (Table 1), then compared results to published clinical data (49, 53, 54).

Specifically, for calibration of immune response to infection, we digitized average longitudinal viral load and immune variable data (naive CD4^+^ T cells and CD8^+^ T cells, effector CD4^+^ T cells and CD8^+^ T cells, type I and type II IFNs, IL-6, and neutralizing antibody titers) for moderately infected COVID-19 patients (*N* = 80 patients) from Lucas et al. (47). Moderately infected patients were chosen over severely infected ones for the lack of pharmacological intervention in the former, thereby allowing the calibration of purely immune response effects on containing infection. Further, for the calibration of immune response to mRNA-based COVID-19 vaccines in healthy individuals, we extracted average longitudinal immune response data (neutralizing antibody titer, effector CD4^+^ T cells and CD8^+^ T cells) to 2 doses of Pfizer-BioNTech COVID-19 mRNA vaccine (*N* = 31 individuals) from Collier et al. (49). The 2 doses were given 21 days apart, with immune response measured 2 to 4 weeks (3 weeks average) after second dose, 6 months after first dose, and 8 months after first dose. From the same study, average immune response data (neutralizing antibody titer) for 2 doses of Moderna COVID-19 mRNA vaccine (21 days apart) were also extracted for model validation (*N* = 22 patients, see below). For calibration of vaccine immune response in cancer patients undergoing antineoplastic treatment, longitudinal antibody titer data (*N* = 63 patients receiving chemotherapy, *N* = 16 patients receiving immunotherapy) following 2 doses of Pfizer-BioNTech COVID-19 mRNA vaccine were extracted from Peeters et al. (52). The 2 doses were given 21 days apart, and immune response was measured at the time of second dose and 7 and 28 days after second dose. Additional data for model validation (antibody titer kinetics) following 2 and 3 doses of Pfizer-BioNTech COVID-19 mRNA vaccine in healthy individuals was obtained from Bayart et al. (*N* = 158 patients) (53) and Papazisis et al. (*N* = 110 patients) (54), respectively. In Bayart et al., the 2 doses were given 21 days apart, and the immune response was measured 14, 28, 42, 56, 90, and 180 days following the first dose. In Papazisis et al., 2 doses were given 21 days apart, following which a third dose was given 9 months after the second dose; immune response was measured 2 weeks after the first dose, then 2 weeks, 3 months, 6 months, 9 months, and 12 months after the second dose. To account for the uncertainty in parameter estimation during model calibration, model predictions were accompanied by 90% prediction interval obtained through 10,000 simulation runs of the model, where each simulation was obtained for a unique set of parameter values drawn through LHS. Note that for LHS, all parameter values were chosen from within a ±10% range of the baseline values estimated during model calibration with healthy individuals.

### Vaccine efficacy estimation.

In accordance with the literature (14, 55), we used the plasma levels of neutralizing IgG anti-spike antibodies against SARS-CoV-2 as predictors of vaccine efficacy (i.e., correlate of protection against SARS-CoV-2). For this, we characterized an empirical correlation between neutralizing antibody titer — Ab(*t*) — and vaccine efficacy — *V*_eff_(*t*) — based on clinical data from the literature (58). The following Michaelis-Menten function was thus used:


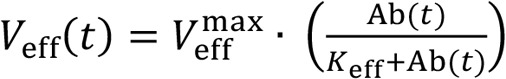
, (Equation 1)

where 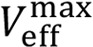 is the maximum possible efficacy of antibody and *K*_eff_ is the Michaelis constant for vaccine efficacy.

As shown in Supplemental Figure 1 (solid blue curve), the above function is in excellent agreement with the clinical data, giving an estimate for *K*_eff_ = 18.95 U/mL (i.e., 50% efficacy) and 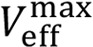
= 92.47%. While only 50% efficacy is necessary to obtain approval for clinical use of vaccines (59), our analysis is based on a more stringent threshold to ensure protection in the majority of recipients. According to Goldblatt et al. (58), the average plasma antibody titer for various COVID-19 vaccines to be protective against WT SARS-CoV-2 is 154 U/mL, which corresponds to a vaccine efficacy of ~82.3% on the Michaelis-Menten curve. Therefore, 154 U/mL was used as a threshold to differentiate protected versus nonprotected individuals in our analyses. *Note that in this work we assume that individuals with antibody titer above the protection threshold are fully protected, while the ones below are fully at risk of infection* (60).

Of note, for the VOCs, the protective threshold was corrected for by using the binding score (Ab_escape_) of the antibodies obtained from the literature (61). This was done to penalize the Michaelis constant such that the potency of the antibodies to neutralize the VOCs was reduced. For this, the previous function was modified to:


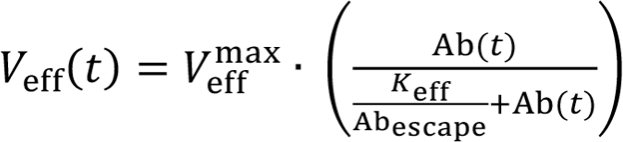
, (Equation 2)

where Ab_escape_ is a dimensionless binding score (Ab_escape_ ϵ [0,1]) obtained from Greaney et al. (61) that quantifies antibody escape, i.e., the inability of antibodies to neutralize the virus due to inefficient binding. As per Greaney et al., the value of Ab_escape_ for WT is 1 and that for the VOC studied here (i.e., OM) is 0.2, indicating that the strength of binding of antibodies to OM is 5 times lesser than that for WT. As a result, 770 U/mL (i.e., 5 times of 154 U/mL) was the estimated threshold of protection against OM, corresponding to 82.3% vaccine efficacy (based on dotted orange curve in Supplemental Figure 1). Of note, the above calculations assume that the mutations in the RBD or NTD of SARS-CoV-2 spike protein negatively affect the binding affinity of antibodies (62), which implies that to obtain a similar protection against OM, or other VOCs, a higher antibody titer is necessary.

### Sensitivity analysis.

To evaluate the relative effect of model parameters on antibody titer following an injection of mRNA-based COVID-19 vaccine, we performed GSA and LSA with parameters of interest. For this, model parameters were perturbed from their baseline values, and the effect of parameter perturbation on model output of interest was quantified (i.e., antibody titer). First, to rank order the parameters for their relative importance in determining antibody titer following vaccination, GSA was performed where all model parameters were *simultaneously* perturbed over a uniformly distributed range of ±50% around the baseline parameter values (obtained from model calibration), except parameter *f* that was perturbed between the values of 0.1–1 (left half-Gaussian distribution); area under the antibody concentration kinetics curve from 0 to 15 days postinjection was calculated for each simulation (i.e., for a given combination of parameter values). Note, to comprehensively investigate the vast multiparameter space (22 parameters), yet to minimize the number of simulations, LHS was used to obtain 10,000 combinations of parameter values, and 10 such replicates were obtained, based on our previously developed workflows (41, 42). Multivariate linear regression analysis was then performed on every replicate, and regression coefficients were determined as a quantitative measure of parameter SI. A distribution of regression coefficients (or SI) was obtained for each parameter, and 1-way ANOVA with Tukey’s test was used to rank order the parameters in terms of their sensitivity, such that a higher SI represents a greater influence on model output (i.e., AUC of antibody titer).

Next, to evaluate the correlation between parameter perturbations and change in antibody titer, LSA was performed, where parameters were perturbed individually at 100 linearly spaced levels over a uniformly distributed range of ±50% around the baseline parameter values (obtained from model calibration). The corresponding change in AUC of antibody titer (from 0 to 15 days postinjection) with respect to parameter perturbation was calculated with the following formula for SI:


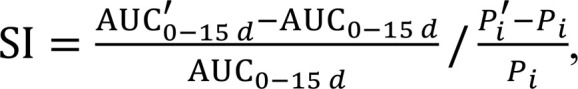
, (Equation 3)

where AUC_0–15 d_ is the AUC of antibody titer under baseline conditions from 0–15 days following injection, AUC*′*_0–15 d_ is the AUC of antibody titer under parameter perturbation condition, *P_i_* is the baseline value of parameter *i*, and *P′_i_* is the perturbed value of parameter *i*.

### Virtual patient cohort design.

To perform population-scale numerical experiments, 2 types of patient populations were generated, namely cohort A and cohort B, as described below.

### Cohort A.

A virtual cohort of 10,000 individuals was generated using LHS (63–65) from 12 parameter distributions (Supplemental Figure 2), such that each individual of the cohort varied in terms of immune health status defined by *f*, underlying biology (characterized by the high-ranking parameters of GSA, i.e., top 10 parameters), and vaccination schedule. The chosen range for the parameter values was such that the *f* parameter varied between 0.5 and 1 (left half-Gaussian distribution with mode equal to 1 and SD equal to 15% of the mode, Supplemental Figure 2C), while the other biological parameters were normally distributed with a mean equal to the baseline parameter value and 1 SD equal to 5% of the mean value (Supplemental Figure 2, D–L); the dosing schedules varied between 2 weeks and 8 weeks for the second dose (continuous uniform distribution, Supplemental Figure 2A) and between 5 months and 9 months for the first booster dose (i.e., third dose; continuous uniform distribution, Supplemental Figure 2B). Note that to reflect the distribution of immune health status and biological variability in a population realistically, i.e., *majority* of the population comprising healthy individuals, Gaussian distributions with limited variance were chosen over uniform distributions for LHS.

### Cohort B.

Three virtual subcohorts of 10,000 individuals each to represent healthy, mildly immunocompromised, and highly immunocompromised individuals were generated through LHS. The range of *f* values used to represent immune health status was *f* = 0.9 to 1 for healthy (continuous uniform distribution), *f* = 0.7 to 0.9 for mildly immunocompromised (continuous uniform distribution), and *f* = 0.5 to 0.7 for highly immunocompromised individuals (continuous uniform distribution). Also, biological variability was included in each subcohort by LHS of the relevant biological parameters (identified through GSA, same as used for cohort A), assumed to be normally distributed with a mean equal to the baseline parameter value and 1 SD equal to 5% of the mean value. For each subcohort, we tested 100 dosing schedules ranging from 2 to 8 weeks (after the first dose) for the second dose (continuous uniform distribution), 0.5 to 9 months (after the second dose) for the first booster (i.e., third dose) (continuous uniform distribution), and 1 to 9 months (after the first booster) for the second booster (i.e., fourth dose; continuous uniform distribution) for their effect on continuity of protection against OM (i.e., vulnerability to breakthrough infection due to mutants).

### Vulnerability kinetics and vaccine dosing schedule optimization.

To study the temporal evolution and quantify the vulnerability to breakthrough infections at the population scale, we calculated a vulnerability kinetics curve in our numerical experiments (as shown in Figure 6C). From the vaccine efficacy calculation (based on Supplemental Figure 1), on a given day, the fraction of simulated individuals below the protection threshold for OM or WT (i.e., <82.3% efficacy) was calculated to obtain the population fraction that is at a high risk of infection. Performing this calculation daily for the entire simulation period gave us the curve shown in Figure 6C, referred to as the vulnerability kinetics curve. Subsequently, we calculated the AUC as a measure of total vulnerability to breakthrough infections, which was then used as a metric for optimizing dosing schedules to impart prolonged protection against OM, as discussed below. Note that we do not use epidemiological principles to model the spread of infection or risk of exposure among the individuals of virtual cohorts, given that the goal of our work is to evaluate the effect of dosing schedule optimization in maintaining protection in already exposed individuals.

To optimize the timing of the second dose, immune response kinetics for each virtual individual (cohort B) was simulated for up to 150 days after the first dose (given on day 0). From the corresponding antibody concentration kinetics, the vaccine efficacy kinetics for OM was computed using Equation 2. Subsequently, we estimated the vulnerability to breakthrough infections over time. From the vulnerability kinetics plot, the AUC_0–150 d_ was calculated using the trapezoidal method. After calculating the AUC_0–150 d_ for 100 dosing schedules (ranging from 2 to 8 weeks for the 3 cohorts), we identified the schedules that led to a minimum in the 3 subpopulations, which translates to a minimized vulnerability to breakthrough infections resulting from OM.

Next, using the optima found in the previous step, we repeated the process to identify the optimal timing for the first booster (third dose) in the 3 cohorts. In this case, the total simulated time was 500 days. Thus, the AUC_0–500 d_ was calculated from the breakthrough infection vulnerability kinetics plots to identify the minima. Finally, using the optimal dosing schedules for the second dose and first booster (third dose), we estimated the optimal timing for the second booster (fourth dose) using the same process as described before. In this case, the total simulation time was 700 days.

### Statistics.

Statistical analyses were performed in MATLAB R2018a. Data are presented as mean ± SD, and model predictions are accompanied by 90% predictions intervals.

### Study approval.

Only published clinical data were used; hence, the study did not require any institutional approval.

### Data availability.

The model code is available from the authors upon reasonable request. Values for all data points found in graphs can be found in the Supporting Data Values file.

## Author contributions

PD conceived and supervised the study and also developed the mathematical model. CS curated the data and performed modeling analyses and simulations. PD, CS, ZW, JRR, SC, and VC designed numerical experiments. PD, CS, DIS, CM, JW, HDS, RP, and WA assisted with biological and clinical interpretation and contextualization of the mathematical modeling results. All authors contributed to manuscript writing and editing.

## Supplementary Material

Supplemental data

Supporting data values

## Figures and Tables

**Table 1:**
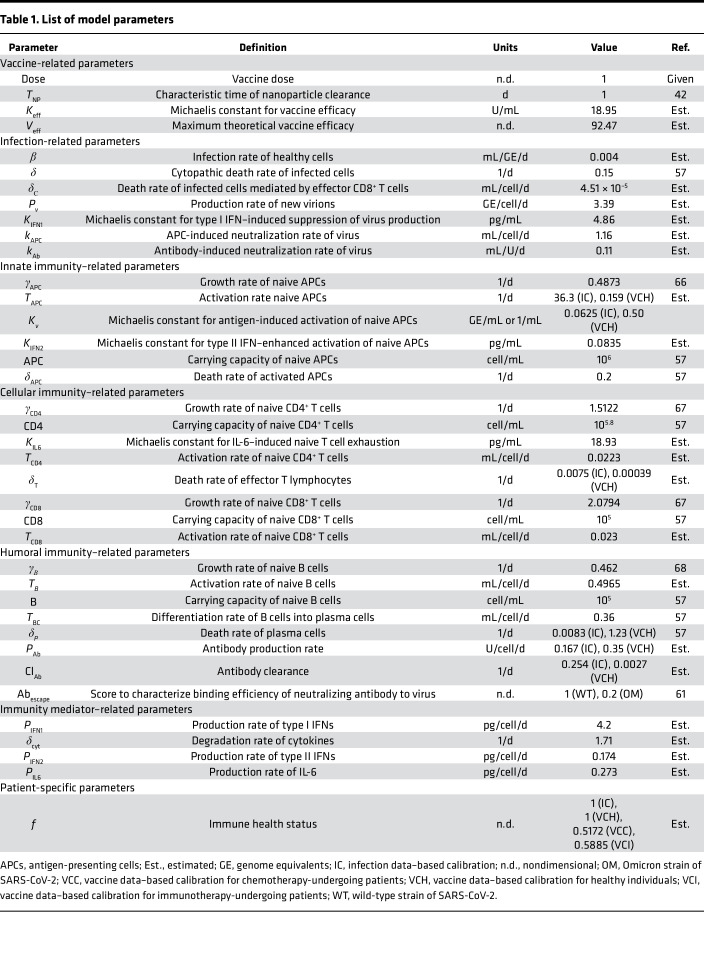
List of model parameters

## Version 1 05/25/2023

In-Press Preview

## Version 2 07/10/2023

Electronic publication
