# Supplementary material for: A modeling-based approach to optimize COVID-19 vaccine dosing schedules for improved protection
Source: JCI Insight. 2023 Jul 10;8(13):e169860. doi: 10.1172/jci.insight.169860 (PMC10371350; doi:10.1172/jci.insight.169860)
Supplement: Supplemental data [file jciinsight-8-169860-s108.pdf]

A modeling-based approach to optimize COVID-19 vaccine dosing schedules for improved protection

Prashant Dogra, Carmine Schiavone, Zhihui Wang, Javier Ruiz-Ramírez, Sergio Caserta, Daniela I. Staquicini, Christopher Markosian, Jin Wang, H. Dirk Sostman, Renata Pasqualini, Wadih Arap, Vittorio Cristini

Supplementary Information

Methods

S1. Mathematical model description

At the site of vaccination, nanoparticles carrying the mRNA of SARS-CoV-2 spike protein are endocytosed into myocytes, leading to the translation and expression of spike protein on myocytes (1). Given that the timescale of drug delivery (intramuscular injection) and mRNA translation is much shorter (< 1 hour) (2) than that of the vaccine-induced immune response (days to weeks) (3), we assumed that the variable $C_a(t)$ represents the concentration of vaccine-induced spike protein in the muscle cells that can trigger the immune response via antigen-presenting cells (APCs).

Concentration kinetics of the exogenously administered antigen (via vaccine) in muscle cells ($\boldsymbol{C_a(t)}$)

$$C_a(t) = \sum_{\tau_i \text{ in } S^T} \left(\text{Dose} \cdot e^{-\frac{1}{2}\left(\frac{t-\tau_i}{T_{\text{NP}}}\right)^2} \right), \quad \textbf{(S1)}$$

where Dose indicates the dimensionless dose of the antigen administered via the vaccine. The concentration of the spike protein $C_a(t)$ is described by the sum of Gaussians centered at τ_i, which represents the day on which a vaccine dose is injected out of the set of doses indicated by S^T. T_{NP} is the characteristic time of clearance of the antigen-carrying nanoparticle (NP) from the body (4), estimated based on NP diameter of 100 nm for mRNA vaccines (5).

The population of naïve (or immature) APCs is maintained through continuous regeneration and presumably maintained at a steady state. Thus, we used a logistic growth term to include this contribution, where γ_{APC} is the exponential growth rate, and $\overline{\text{APC}}$ is the carrying capacity of the APC population. Naïve APCs at the site of expression of spike proteins recognize, process, and present the antigen via major histocompatibility complex (MHC) during differentiation into activated APC (APC^*) at a rate T_{APC} as they migrate towards the lymphoid tissue. The APC activation process is proportional to the antigen load ($\text{Ag}(t)$), which can be derived either from the vaccine or natural

infection and is either equal to $C_a(t)$ or the viral load $V(t)$ in the case of vaccination or infection, respectively, with K_v being the Michaelis constant for antigen-induced activation of naïve APCs.

Equation for the naïve APC density at the site of vaccination or natural infection ($\mathbf{APC}(\boldsymbol{t})$)

$$\frac{d\mathrm{APC}(t)}{dt} = \overbrace{\gamma_{\mathrm{APC}} \cdot \mathrm{APC}(t) \cdot \left(1 - \frac{\mathrm{APC}(t)}{\overline{\mathrm{APC}}}\right)}^{Regeneration} - \overbrace{T_{\mathrm{APC}} \cdot \mathrm{APC}(t) \cdot \frac{\mathrm{Ag}(t)}{K_v + \mathrm{Ag}(t)}}^{Activation}, \quad \mathrm{APC}(0) = \overline{\mathrm{APC}} \qquad \textbf{(S2)}$$

Activated APCs are primarily responsible for the induction of the adaptive immune response, and their population is determined by the activation of naïve APCs, which we discussed in Eq. S2, and a death term determined by the death rate constant δ_{APC} of activated APCs.

Equation for the activated APC density ($\mathbf{APC}^*(\boldsymbol{t})$)

$$\frac{d\mathrm{APC}^*(t)}{dt} = \overbrace{T_{\mathrm{APC}} \cdot \mathrm{APC}(t) \cdot \frac{\mathrm{Ag}(t)}{K_v + \mathrm{Ag}(t)}}^{Activation} - \overbrace{\delta_{\mathrm{APC}} \cdot \mathrm{APC}^*(t)}^{Death}, \qquad \mathrm{APC}^*(0) = 0 \qquad \textbf{(S3)}$$

Activated APCs migrate from the site of vaccination or natural infection to the lymphoid tissue to interact with naïve T-cells (CD8+ or CD4+) and transform them into their active or effector forms. Alternatively, naïve B-cells are activated by the binding of soluble antigens, which however in the current model is replaced by binding to active APCs, given that the density of active APCs is dependent on antigen load in the body. For the naïve cells, population density is determined by cell regeneration and cell activation, where we used a logistic growth term with γ_{CD4}, γ_{CD8}, and γ_{B} as the growth rates of naïve forms of CD4+ T-cells, CD8+ T-cells, and B cells, respectively; $\overline{\mathrm{CD4}}$, $\overline{\mathrm{CD8}}$, and $\overline{\mathrm{B}}$ are the carrying capacities of the corresponding cell populations. The activation term has second-order kinetics and is proportional to the product of active APC density and the corresponding naïve cell density; $T_{\mathrm{CD4}}, T_{\mathrm{CD8}}$ and T_{B} are the activation rates of lymphocytes indicated by the subscript. Activation of T-cells is amplified by the presence of type-II interferons ($\mathrm{IFN2}(t)$) secreted by

activated T- cells (6), with possible saturation effects. Thus, we used a Michaelis-Menten term to model this process in which K_{IFN2} is the Michaelis constant of type-II interferon effects.

Of note, in our model we have included a dimensionless coefficient $f \in [0,1]$ that represents an immunosuppression factor to modulate the carrying capacity (i.e., homeostasis levels) of the naïve immune cell population to model immunocompromised subjects, such that $f = 1$ in healthy individuals, and $f < 1$ in immunocompromised patients. Since our model is calibrated for patients who are immunocompromised due to anticancer therapy, immunosuppression in our model is characterized by T- and B-cell deficiency, which is one of the important immunological effects observed due to disruption of hematopoiesis leading to myelosuppression in patients undergoing anticancer therapy (7-11). Also, in the case of naïve CD4+ and CD8+ T-cells we have included the ability of interleukin-6 (IL-6) to cause T-cell exhaustion (12) by including an additional term that limits the carrying capacity of these cells. This term uses the concentration of IL-6 in a Michaelis-Menten function, where K_{IL6} is the Michaelis constant for IL-6 effects.

Equation for the naïve CD4+ T-cell density ($\mathbf{CD4}(\boldsymbol{t})$)

$$\frac{d\mathrm{CD4}(t)}{dt} = \overbrace{\gamma_{\mathrm{CD4}} \cdot \mathrm{CD4}(t) \cdot \left(1 - \frac{\mathrm{CD4}(t)}{\underbrace{f \cdot \overline{\mathrm{CD4}} \cdot \left(1 - \frac{\mathrm{IL6}(t)}{K_{\mathrm{IL6}} + \mathrm{IL6}(t)}\right)}_{T-cell\ exhaustion}}\right)}^{Regeneration} - \overbrace{T_{\mathrm{CD4}} \cdot \mathrm{APC}^*(t) \cdot \mathrm{CD4}(t) \cdot \left(1 + \frac{\mathrm{IFN2}(t)}{K_{\mathrm{IFN2}} + \mathrm{IFN2}(t)}\right)}^{Activation}, \quad \mathrm{CD4}(0) = f \cdot \overline{\mathrm{CD4}} \qquad \textbf{(S4)}$$

Equation for the effector CD4+ T-cell density ($\mathbf{CD4}^*(\boldsymbol{t})$)

$$\frac{d\mathrm{CD4}^*(t)}{dt} = \overbrace{T_{\mathrm{CD4}} \cdot \mathrm{APC}^*(t) \cdot \mathrm{CD4}(t) \cdot \left(1 + \frac{\mathrm{IFN2}(t)}{K_{\mathrm{IFN2}} + \mathrm{IFN2}(t)}\right)}^{Activation} - \overbrace{\delta_T \cdot \mathrm{CD4}^*(t)}^{Death}, \quad \mathrm{CD4}^*(0) = 0 \qquad \textbf{(S5)}$$

where δ_T is the death rate of effector T-cells.

Equation for the naïve CD8+ T-cell density ($\mathbf{CD8}(\boldsymbol{t})$)

$$\frac{d\mathrm{CD8}(t)}{dt} = \overbrace{\gamma_{\mathrm{CD8}} \cdot \mathrm{CD8}(t) \cdot \left(1 - \frac{\mathrm{CD8}(t)}{\underbrace{f \cdot \overline{\mathrm{CD8}} \cdot \left(1 - \frac{\mathrm{IL6}(t)}{K_{\mathrm{IL6}} + \mathrm{IL6}(t)}\right)}_{T-cell\ exhaustion}}\right)}^{Regeneration} -$$

$$\overbrace{T_{\mathrm{CD8}} \cdot \mathrm{APC}^*(t) \cdot \mathrm{CD8}(t) \cdot \left(1 + \frac{\mathrm{IFN2}(t)}{K_{\mathrm{IFN2}} + \mathrm{IFN2}(t)}\right)}^{Activation}, \qquad \mathrm{CD8}(0) = f \cdot \overline{\mathrm{CD8}} \qquad \mathbf{(S6)}$$

Equation for the effector CD8+ T-cell density ($\mathbf{CD8}^*(\boldsymbol{t})$)

$$\frac{d\mathrm{CD8}^*(t)}{dt} = \overbrace{T_{\mathrm{CD8}} \cdot \mathrm{APC}^*(t) \cdot \mathrm{CD8}(t) \cdot \left(1 + \frac{\mathrm{IFN2}(t)}{K_{\mathrm{IFN2}} + \mathrm{IFN2}(t)}\right)}^{Activation} - \overbrace{\delta_T \cdot \mathrm{CD8}^*(t)}^{Death},$$

$$\mathrm{CD8}^*(0) = 0 \qquad \mathbf{(S7)}$$

Equation for the naïve B cell density ($\boldsymbol{B}(\boldsymbol{t})$)

$$\frac{dB(t)}{dt} = \overbrace{\gamma_{\mathrm{B}} \cdot B(t) \cdot \left(1 - \frac{B(t)}{f \cdot \bar{B}}\right)}^{Regeneration} - \overbrace{T_{\mathrm{B}} \cdot \mathrm{APC}^*(t) \cdot B(t)}^{Activation}, \qquad B(0) = f \cdot \bar{B} \quad \mathbf{(S8)}$$

where T_{B} is the transition rate of naïve B cells into their activated form.

Of note, the activated B cells differentiate into antibody-secreting plasma cells upon interaction with effector CD4+ T-cells. We modeled this interaction using second-order kinetics, where T_{BC} is the differentiation rate of B cells into plasma cells.

Equation for the activated B cell density ($B^*(t)$)

$$\frac{dB^*(t)}{dt} = \overbrace{T_{\mathrm{B}} \cdot \mathrm{APC}^*(t) \cdot B(t)}^{Activation} - \overbrace{T_{\mathrm{BC}} \cdot \mathrm{CD4}^*(t) \cdot B^*(t)}^{Differentiation}, \qquad B^*(0) = 0 \qquad \textbf{(S9)}$$

where T_{BC} is the differentiation rate of B cells into plasma cells.

Equation for the plasma cell density ($P(t)$)

$$\frac{dP(t)}{dt} = \overbrace{T_{\mathrm{BC}} \cdot \mathrm{CD4}^*(t) \cdot B^*(t)}^{Differentiation} - \overbrace{\delta_P \cdot P(t)}^{Death}, \qquad P(0) = 0 \qquad \textbf{(S10)}$$

where δ_P is the death rate of plasma cells.

Virus-neutralizing antibodies are secreted by plasma cells, such that their rate of production, characterized by the first-order rate constant P_{Ab}, is proportional to the plasma cell density. The antibodies secreted into the plasma are then cleared at a rate $\mathrm{Cl}_{\mathrm{Ab}}$, which is a lumped phenomenological parameter characterizing the various antibody clearance mechanisms.

Equation for the neutralizing antibody concentration (Ab(t))

$$\frac{d\mathrm{Ab}(t)}{dt} = \overbrace{P_{\mathrm{Ab}} \cdot P(t)}^{Production} - \overbrace{\mathrm{Cl}_{\mathrm{Ab}} \cdot \mathrm{Ab}(t)}^{Clearance}, \qquad \mathrm{Ab}(0) = 0 \qquad \textbf{(S11)}$$

Following vaccination or natural infection, the immune system produces different cytokines to regulate cellular activation and differentiation, as discussed above. In the specific case of SARS-CoV-2, it has been shown that type-I and type-II interferons, and IL-6 are the relevant immunoregulatory elements (13, 14). Each cytokine has a unique source and key role in the immune response. For instance, type-I interferons (IFN1(t)), secreted by virus-infected cells or vaccine-affected cells, lowers the production of new virions by infected cells (6); type-II interferon (IFN2(t)),

produced by effector CD4+ and effector CD8+ T-cells, accelerates the differentiation of naive T-cells into their effector form in a positive feedback loop fashion (6); and IL-6, secreted by effector CD4+ T-cells, effector CD8+ T-cells, and active APCs, tends to exhaust naïve CD4+ and CD8+ T-cell populations (12). The rate of change of cytokine concentration was modeled using production term and degradation terms, where production and degradation are modeled as first-order processes, with degradation characterized by a common degradation rate constant δ_{cyt}.

Equation for the type-I interferon concentration (IFN1(t))

$$\frac{d\text{IFN1}(t)}{dt} = \overbrace{P_{\text{IFN1}} \cdot \big(I(t) + C_a(t)\big)}^{Production} - \overbrace{\delta_{\text{cyt}} \cdot \text{IFN1}(t)}^{Degradation}, \qquad \text{IFN1}(0) = 0 \quad \textbf{(S12)}$$

where P_{IFN1} is the production rate of type-I interferon.

Equation for the type-II interferon concentration (IFN2(t))

$$\frac{d\text{IFN2}(t)}{dt} = \overbrace{P_{\text{IFN2}} \cdot \big(\text{CD4}^*(t) + \text{CD8}^*(t)\big)}^{Production} - \overbrace{\delta_{\text{cyt}} \cdot \text{IFN2}(t)}^{Degradation}, \qquad \text{IFN2}(0) = 0 \quad \textbf{(S13)}$$

where P_{IFN2} is the production rate of type-II interferon.

Equation for the interleukin-6 concentration (IL6(t))

$$\frac{d\text{IL6}(t)}{dt} = \overbrace{P_{\text{IL6}} \cdot (\text{CD4}^*(t) + \text{CD8}^*(t) + \text{APC}^*(t))}^{Production} - \overbrace{\delta_{\text{cyt}} \cdot \text{IL6}(t)}^{Degradation}, \qquad \text{IL6}(0) = 0 \quad \textbf{(S14)}$$

where, P_{IL6} is the production rate of IL-6.

The entire immune cascade can be triggered either by a vaccine (as we have already elaborated), or through natural infection. In the latter case, healthy susceptible cells are transformed into infected

cells by the virus, followed by production of new viral particles by the infected cells. With the intent to develop a generalized mathematical model capable of simulating immune response to vaccines as well as infections, we incorporate the infection process into our model, with the respiratory tract as a representative site. For this, we used a target cell limited model of acute viral infection (15), as described by the equations below:

Equation for the healthy respiratory epithelial cell density ($\boldsymbol{H(t)}$)

$$\frac{dH(t)}{dt} = -\overbrace{\beta \cdot H(t) \cdot V(t)}^{Infection}, \qquad H(0) = H_0 \qquad \textbf{(S15)}$$

where β is the viral infectivity rate, $V(t)$ is the viral load density in the respiratory tract, and H_0 is the initial density of healthy cells.

Equation for the density of infected cells in the respiratory tract epithelium ($\boldsymbol{I(t)}$)

$$\frac{dI(t)}{dt} = \overbrace{\beta \cdot H(t) \cdot V(t)}^{Infection} - \overbrace{\delta \cdot I(t)}^{Cytopathic\ death} - \overbrace{\delta_{\mathrm{C}} \cdot I(t) \cdot \mathrm{CD8}^{*}(t)}^{T-cell-mediated\ death}, \qquad I(0) = 0 \qquad \textbf{(S16)}$$

where δ represents the cytopathic death rate of infected cells, δ_{C} is the death rate of infected cells mediated by effector CD8+ T-cells, and $\mathrm{CD8}^{*}(t)$ is the density of effector CD8+ T-cells.

Equation for the viral load density in the respiratory tract ($\boldsymbol{V(t)}$)

$$\frac{dV(t)}{dt} = \overbrace{P_v \cdot I(t) \cdot \underbrace{\left(1 - \frac{\mathrm{IFN1}(t)}{K_{\mathrm{IFN1}} + \mathrm{IFN1}(t)}\right)}_{Viral\ production\ suppression}}^{New\ virion\ production} - \overbrace{k_{\mathrm{Ab}} \cdot V(t) \cdot \mathrm{Ab}(t)}^{Antibody\ neutralization} - \overbrace{k_{\mathrm{APC}} \cdot V(t) \cdot \mathrm{APC}(t)}^{APC-mediated\ clearance},$$

$$V(0) = V_0 \qquad \textbf{(S17)}$$

where P_v represents virion production rate, $\mathrm{IFN1}(t)$ is the concentration of type-I interferons, K_{IFN1} is the Michaelis constant of the virion production suppression factor, k_{Ab} is the antibody-mediated neutralization rate of viruses, $\mathrm{Ab}(t)$ is the antibody concentration in the body, $\mathrm{APC}(t)$ is the density

of naïve APCs in the respiratory tract, k_{APC} is the naïve APC-mediated clearance rate of viruses, and V_0 is the initial viral load at the time of infection.

Results

Antibody titer as a correlate of vaccine efficacy (or protection)

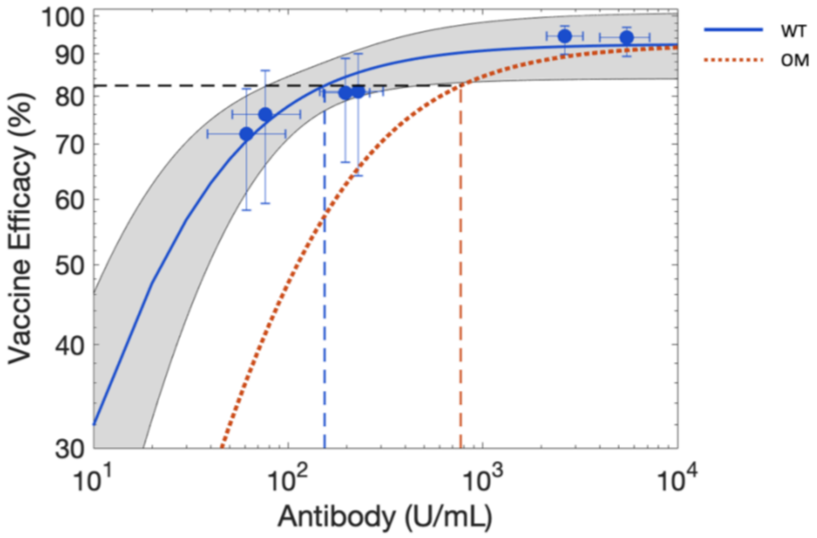

Figure S1. Least squares fitting of Michaelis-Menten function (Equation 1; solid blue curve) to clinical data (blue circles), obtained from Goldblatt et al. (16), for the effect of antibody (IgG) titer on vaccine efficacy against wild type (WT) strain of SARS-CoV-2. Gray band is the 95% confidence interval band. Dotted orange curve represents the corresponding vaccine efficacy prediction (based on Equation 2) for the Omicron (OM) strain of SARS-CoV-2. Error bars indicate 95% confidence intervals for either the antibody level (x-axis) or vaccine efficacy (y-axis). Dashed blue and orange lines denote antibody titer values of 154 U/mL and 770 U/mL, which corresponds to 82.3% vaccine efficacy (dashed black line) against WT and OM, respectively. Note: x- and y-axes are in log scale.

Parameter distributions for virtual cohort generation

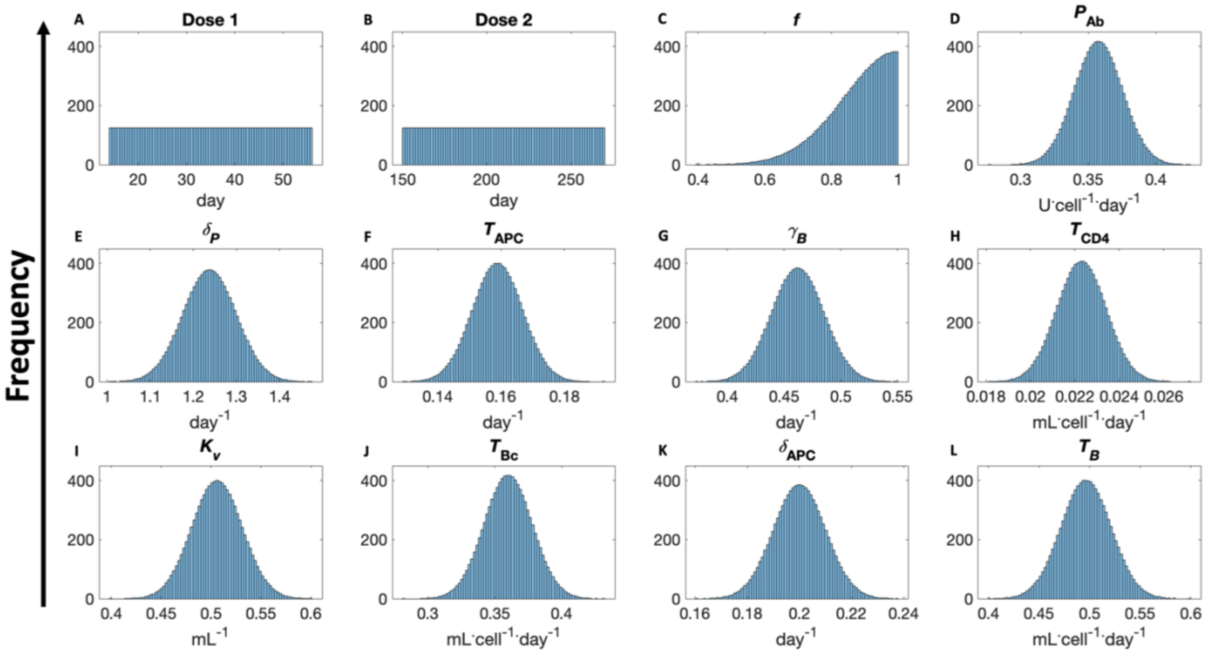

Figure S2. Representative distributions (N = 10,000) of model parameters, identified from GSA as highly sensitive parameters, used for virtual clinical simulations.

Pearson correlation analysis (model calibration)

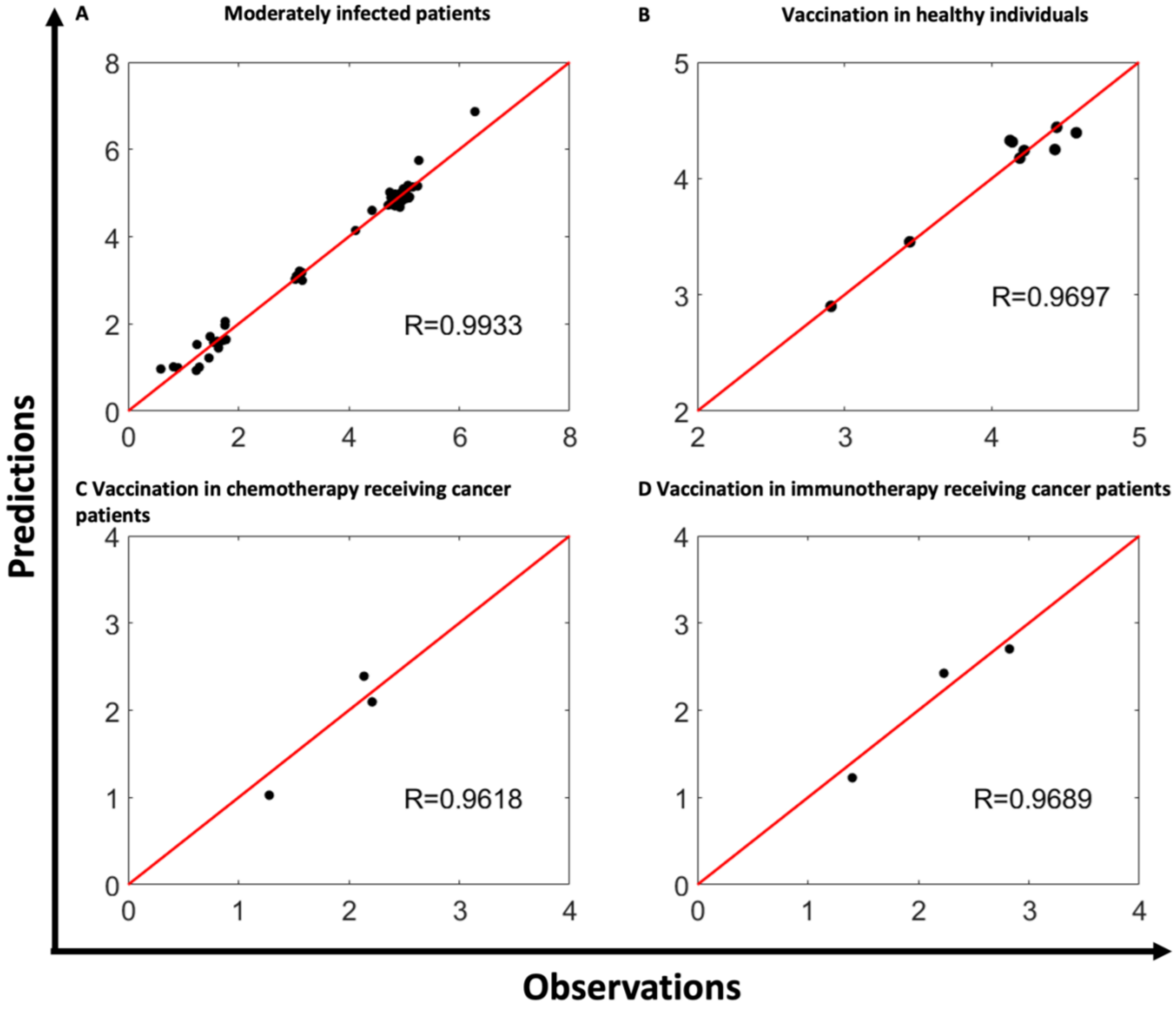

Figure S3. Pearson correlation analysis between model fits and experimental data for immune response dynamics (comprising one or more of the following variables: viral load, naïve and effector CD4+ and CD8+ T-cells, type-I and type-II interferon, IL-6, and neutralizing antibody (IgG)) during **A)** moderate SARS-CoV-2 infection, vaccination in **B**) healthy individuals, **C**) cancer patients receiving chemotherapy, and **D**) cancer patients receiving immunotherapy. *R* value represents Pearson correlation coefficient.

Pearson Correlation analysis (model validation)

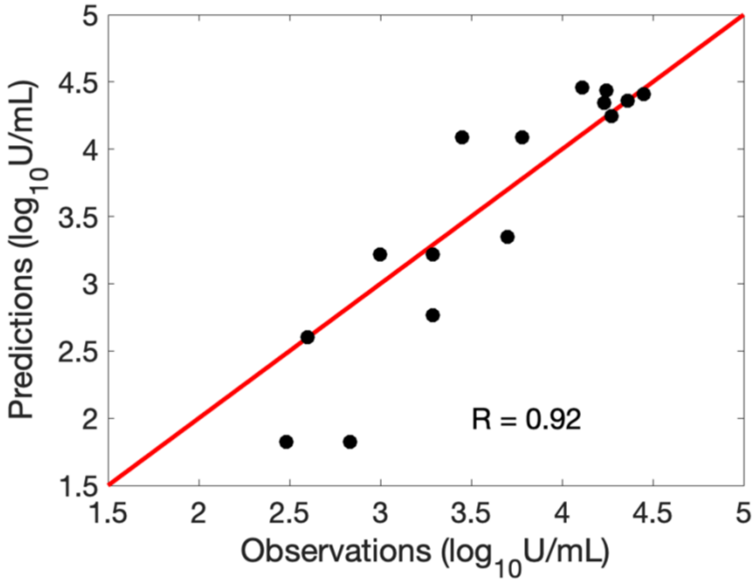

Figure S4. Pearson correlation analysis between model predictions and experimental data pooled from clinical studies on antibody (IgG) response dynamics following two doses of Moderna COVID-19 mRNA vaccine, and two and three doses of Pfizer-BioNTech COVID-19 mRNA vaccine. *R* value represents Pearson correlation coefficient.

Local sensitivity analysis

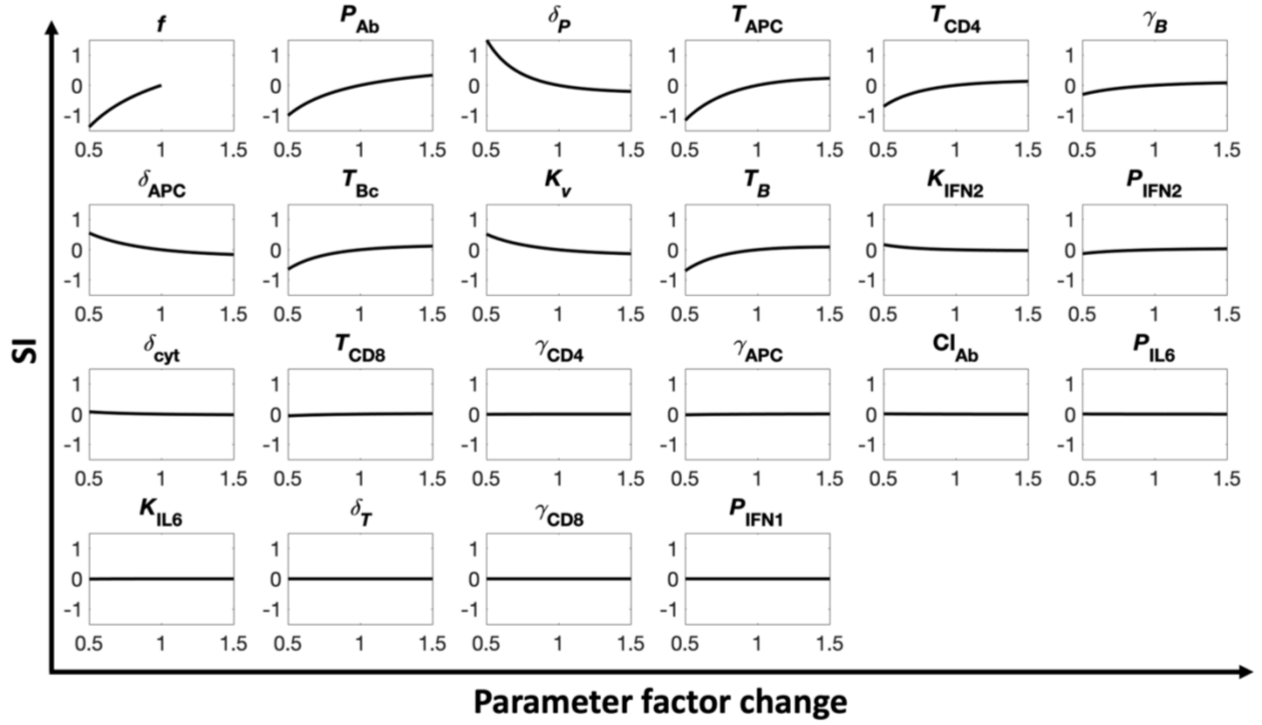

Figure S5. Local sensitivity analysis exhibiting correlation between parameter perturbation and sensitivity index. Parameters were perturbed linearly between $\pm$50% around the baseline value, except f that was perturbed between –50% to baseline.

References

1. Liang F, et al. Efficient Targeting and Activation of Antigen-Presenting Cells In Vivo after Modified mRNA Vaccine Administration in Rhesus Macaques. *Molecular therapy : the journal of the American Society of Gene Therapy*. 2017;25(12):2635-47.
2. Milo R, Phillips R. Cell biology by the numbers: Garland Science; 2015.
3. Cox RJ, Brokstad KA. Not just antibodies: B cells and T cells mediate immunity to COVID-19. *Nature Reviews Immunology*. 2020;20(10):581-2.
4. Dogra P, et al. Translational Modeling Identifies Synergy between Nanoparticle-Delivered miRNA-22 and Standard-of-Care Drugs in Triple-Negative Breast Cancer. *Pharmaceutical Research*. 2022;39(3):511-28.
5. Schoenmaker L, et al. mRNA-lipid nanoparticle COVID-19 vaccines: Structure and stability. *International Journal of Pharmaceutics*. 2021;601:120586.
6. Abbas AK, et al. Basic immunology e-book: functions and disorders of the immune system: Elsevier Health Sciences; 2019.
7. Ahlmann M, Hempel G. The effect of cyclophosphamide on the immune system: implications for clinical cancer therapy. *Cancer Chemother Pharmacol*. 2016;78(4):661-71.
8. Epstein RS, et al. Patient Burden and Real-World Management of Chemotherapy-Induced Myelosuppression: Results from an Online Survey of Patients with Solid Tumors. *Adv Ther*. 2020;37(8):3606-18.
9. Epstein RS, et al. Treatment patterns and burden of myelosuppression for patients with small cell lung cancer: A SEER-medicare study. *Cancer Treatment and Research Communications*. 2022;31:100555.
10. Atwal D, et al. Pembrolizumab-Induced Pancytopenia: A Case Report. *Perm J*. 2017;21:17-004.
11. Ueki Y, et al. Pembrolizumab-induced pancytopenia in a patient with squamous cell lung cancer. *Thoracic Cancer*. 2020;11(9):2731-5.
12. Diao B, et al. Reduction and Functional Exhaustion of T Cells in Patients With Coronavirus Disease 2019 (COVID-19). *Frontiers in Immunology*. 2020;11.
13. Vatansever HS, Becer E. Relationship between IL-6 and COVID-19: to be considered during treatment. 2020;15(12):817-22.
14. Galbraith MD, et al. Specialized interferon action in COVID-19. *Proc Natl Acad Sci U S A*. 2022;119(11).
15. Perelson AS, Ke R. Mechanistic Modeling of SARS-CoV-2 and Other Infectious Diseases and the Effects of Therapeutics. *Clin Pharmacol Ther*. 2021;109(4):829-40.
16. Goldblatt D, et al. Towards a population-based threshold of protection for COVID-19 vaccines. *Vaccine*. 2022;40(2):306-15.
